# The EBV Latent Antigen 3C Inhibits Apoptosis through Targeted Regulation of Interferon Regulatory Factors 4 and 8

**DOI:** 10.1371/journal.ppat.1003314

**Published:** 2013-05-02

**Authors:** Shuvomoy Banerjee, Jie Lu, Qiliang Cai, Abhik Saha, Hem Chandra Jha, Richard Kuo Dzeng, Erle S. Robertson

**Affiliations:** Department of Microbiology and the Tumor Virology Program, Abramson Cancer Center, Perelman School of Medicine at the University of Pennsylvania, Philadelphia, Pennsylvania, United States of America; University of North Carolina at Chapel Hill, United States of America

## Abstract

Epstein-Barr virus (EBV) is linked to a broad spectrum of B-cell malignancies. EBV nuclear antigen 3C (EBNA3C) is an encoded latent antigen required for growth transformation of primary human B-lymphocytes. Interferon regulatory factor 4 (IRF4) and 8 (IRF8) are transcription factors of the IRF family that regulate diverse functions in B cell development. IRF4 is an oncoprotein with anti-apoptotic properties and IRF8 functions as a regulator of apoptosis and tumor suppressor in many hematopoietic malignancies. We now demonstrate that EBNA3C can contribute to B-cell transformation by modulating the molecular interplay between cellular IRF4 and IRF8. We show that EBNA3C physically interacts with IRF4 and IRF8 with its N-terminal domain in vitro and forms a molecular complex in cells. We identified the Spi-1/B motif of IRF4 as critical for EBNA3C interaction. We also demonstrated that EBNA3C can stabilize IRF4, which leads to downregulation of IRF8 by enhancing its proteasome-mediated degradation. Further, si-RNA mediated knock-down of endogenous IRF4 results in a substantial reduction in proliferation of EBV-transformed lymphoblastoid cell lines (LCLs), as well as augmentation of DNA damage-induced apoptosis. IRF4 knockdown also showed reduced expression of its targeted downstream signalling proteins which include CDK6, Cyclin B1 and c-Myc all critical for cell proliferation. These studies provide novel insights into the contribution of EBNA3C to EBV-mediated B-cell transformation through regulation of IRF4 and IRF8 and add another molecular link to the mechanisms by which EBV dysregulates cellular activities, increasing the potential for therapeutic intervention against EBV-associated cancers.

## Author Summary

Interferon regulatory factor (IRF) family members have different roles in context of pathogen response, signal transduction, cell proliferation and hematopoietic development. IRF4 and IRF8 are members of the IRF family and are critical mediators of B-cell development. Enhanced expression of IRF4 is often associated with multiple myeloma and adult T-cell lymphomas. Furthermore, IRF8 can function as a tumor suppressor in myeloid cancers. Epstein-Barr virus (EBV), one of the first characterized human tumor viruses is associated with several lymphoid malignancies. One of the essential antigens, EBV encoded nuclear antigen 3C (EBNA3C), plays a critical role in EBV-induced B-cell transformation. In our study, we now demonstrate that EBNA3C forms a molecular complex with IRF4 and IRF8 specifically through its N-terminal domain. We show that IRF4 is stabilized by EBNA3C, which resulted in downregulation of IRF8 through proteasome-mediated degradation and subsequent inhibition of its tumor suppressive activity. Moreover, si-RNA-mediated inhibition of IRF4 showed a substantial reduction in EBV transformed B-cell proliferation, and also enhanced their sensitivity to DNA-damage induced apoptosis. Therefore, our findings demonstrated that targeted disruption of EBNA3C-mediated differential regulation of IRF4 and IRF8 may have potential therapeutic value for treating EBV induced B-cell malignancies.

## Introduction

Tumor viruses have evolved multiple strategies for modulating the expression of an array of cellular genes to enhance persistence, latency and survival of infected cells. Studies into these strategies have provided several lines of evidence as to the mechanisms of differential gene expression and their deregulation during oncogenesis. Particularly, EBV is responsible for the development of lympho-proliferative diseases manifested in immuno-compromised AIDS patients [1], and is also linked to Burkitt's lymphoma, Hodgkin's lymphoma, B and T cell lymphomas, anaplastic nasopharyngeal carcinoma, and also some forms of gastric carcinomas [2]. Human primary B lymphocytes are the principal target for EBV infection, although the virus has the potential to infect other lymphocytes and epithelial cells [3].

EBV infection transforms primary human B-cells into continuously growing lymphoblastoid cell lines (LCLs) with the establishment of viral latency [4]. Three major types of viral latency have been elucidated with each having their own specific viral gene expression pattern, although other patterns have been described [5]. EBV latency proteins are comprised of EBV nuclear antigens, such as EBNA1, EBNA2, EBNA3A/3, EBNA3B/4, EBNA3C/6 and three latent membrane proteins LMP1, LMP2A and LMP2B [6], [7]. These proteins are all expressed in type III latency, also referred as the growth programme [8]. Six of the EBV encoded latent proteins including, LMP1, EBNA-LP, EBNA1, EBNA2, EBNA3A and EBNA3C were found to be important or critical for B-cell immortalization in vitro [9]. EBNA3C, as demonstrated by genetic analysis using recombinant virus strategies is necessary not only for proficient immortalization of primary human B-cells in vitro [10], but also for the purpose of cell-cycle progression and growth maintenance of EBV-positive lymphoblastoid cells [1]. Interestingly, EBNA3C has the ability to perform both functions as a transcriptional activator and repressor [11], and can interact with a wide range of transcriptional modulators, like PU.1, Spi-B, HDAC1, CtBP, DP103, p300, prothymosin-α, Nm23-H1, SUMO1 and SUMO3 [12]. EBNA3C also plays a critical role in deregulation of mammalian cell-cycle by targeting different cellular onco-proteins and tumor suppressors [13]. Recently, we demonstrated that EBNA3C negatively regulates p53 functions by interacting with ING4 and ING5 [14].

The mammalian interferon regulatory factor (IRF) family of transcription factors were categorized as transcriptional regulators of type I interferon (IFN) and IFN-inducible genes [15]. However, recent studies have revealed that this protein family also plays a vital role in regulation of host defence beyond its function in the IFN-system [16]. The IRF family is comprised of nine members, which include IRF1, IRF2, IRF3, IRF4, IRF5, IRF6, IRF7, IRF8/ICSBP, and IRF9/ISGF3 [17]. IRFs have also been linked to viral induced transformation as EBV-encoded LMP-1 can induce the expression level of IRF7 and its activation through receptor-interacting protein (RIP)-1 and TRAF6 [18]. IRF7 promotes anchorage-independent growth of NIH-3T3 cells, and with LMP-1, it shows an additive effect on the growth of these cells [19]. IRF4, also known as LSIRF, ICSAT, Pip, and Mum1, was cloned independently as the homologous member of the IRF gene family and as an interacting partner of PU.1 [20]. IRF4 is expressed at all developmental stages of B-cell, in mature T-cells, and also in macrophages [21], and the analysis of IRF4 knockout mice revealed that IRF4 is vital for the function and homeostasis of both mature B and T-lymphocytes [22]. IRF4 also has an essential role in T-cell immune responses [23]. In macrophages, IRF4 is essential for TLR signalling [24] and is essential for the expression of surface major histocompatibility complex class II (MHCII) molecule in dendritic cells for antigen presentation [25]. There are some reports which suggest that IRF4 also plays an important role in receptor editing [26]. More recently studies have indicated that IRF4, when overexpressed, functions as an oncoprotein [27]. On the contrary, IRF4 was found down-regulated in some myeloid and early B-lymphoid malignancies [28], and so IRF4 may have different functions in the context of different cell types [29]. IRF4 deficiency facilitates the progression of BCR/ABL-induced B-ALL, while forced expression of IRF4 potently supresses the pathogenesis of BCR/ABL-induced B-ALL [28]. These findings demonstrated that IRF4 can also functions as a tumor suppressor in the myeloid lineage and in early stages of B-cell development.

IRF4 is linked to human T-cell Leukemia virus-induced transformation [30], has oncogenic potential in vitro and can inhibit apoptosis [31]. Recently, it was demonstrated that IRF4 indirectly regulates miR-155, an evolutionarily conserved miRNA, via B-cell integration cluster (BIC) [32]. IRF4 is also important in the pathogenesis of multiple myeloma [33], and is involved in EBV mediated growth transformation of B-lymphocytes [34]. Furthermore, IRF4 is also known to be involved in regulation of EBV-mediated cell growth by down-regulating the expression level of IRF5, another pro-apoptotic member of interferon regulatory factors [35]. This report now shows that IRF4 can play a crucial role in modulating the activities of other critical IRF transcription factors in EBV transformed cells.

Interferon regulatory factor 8 (also known as IFN consensus sequence-binding protein/IRF8) is another potent transcription factor belonging to the IRF family [36]. Expression of IRF8 is generally seen in hematopoietic cells, including monocytes, macrophages and the subsets of lymphocytes [37]. In case of late myeloid differentiation, IRF8 plays an important role in the commitment to macrophage differentiation and is essential for the function of mature macrophages [38]. In contrast, IRF8-null mice show noticeable clonal expansion of undifferentiated granulocytes and macrophages, which often progress to a chronic myelogenous leukemia (CML) like syndrome in these animals [39], [40]. Notably, IRF8 expression was found dramatically decreased in patients with CML and acute myeloid leukemia (AML) [41]. These studies suggested that IRF8 plays a crucial role in regulation of leukemogenesis and functions as a tumor suppressor of certain myeloid malignancies. The molecular mechanism involved in the control of leukemogenesis by IRF8 is yet to be delineated. However, IRF8 deficiency in hematopoietic cells leads to declined spontaneous apoptosis and enhanced resistance to the extrinsic apoptotic induction [42]. These results provide clues as to another potential branch in regulation of critical cellular regulatory components by EBNA3C.

In this current study, we now provide the first evidence linking EBNA3C to differential regulation of IRF4 and IRF8 and show that they can contribute to B cell transformation. Interestingly, our work demonstrated a molecular interplay between IRF4/IRF8 and EBNA3C. Detailed mapping revealed that 29 residues within the N-terminal domain of EBNA3C interact with IRF4 and IRF8 and this interaction led to stabilization of IRF4 by inhibiting its Ub-proteasome-mediated degradation and enhancing the degradation of IRF8. We also found that a Spi-1/B-like motif within the C-terminal domain of IRF4 plays a critical role in binding to EBNA3C. Additionally, RNA interference based strategy to knock-down endogenous IRF4 showed a significant reduction in EBV transformed cell proliferation, as well as increased sensitivity to etoposide-induced apoptosis. We also demonstrated significant down-modulation of IRF4 targeted proteins such as, c-Myc, Cyclin B1 and Cyclin-dependent kinase 6 (CDK6) upon EBNA3C or IRF4 knockdown in lymphoblastoid cells. These results imply that EBNA3C mediated activation of IRF4 and its downstream signalling may deregulate important cellular functions which include proliferation, apoptosis, and cell-cycle which favour the development of B-cell lymphomas. These findings provide important clues to yet another fundamental role of EBNA3C in contributing to EBV-mediated B-cell oncogenesis via differential regulation of IRF4 and IRF8.

## Results

### EBV essential antigen EBNA3C differentially regulates IRF4 and IRF8

IRFs are important contributors to pathogenesis associated with human malignancies [15]. IRF4 is associated with enhanced pathogenesis of EBV-mediated growth transformation of B-lymphocytes, and has been shown to play a primary role in cell proliferation of multiple myeloma [43], [44]. IRF8 has been characterized as a major transcription factor in the IRF family of proteins [45], and its levels are dramatically decreased in CML and myeloid leukemia cells [46]. Here we examined whether IRF4 and IRF8 are potentially involved in EBV mediated B cell transformation as proteomic analysis of EBNA3C complexes isolated from LCLs identified IRF4 and IRF8.

**Figure 1:**
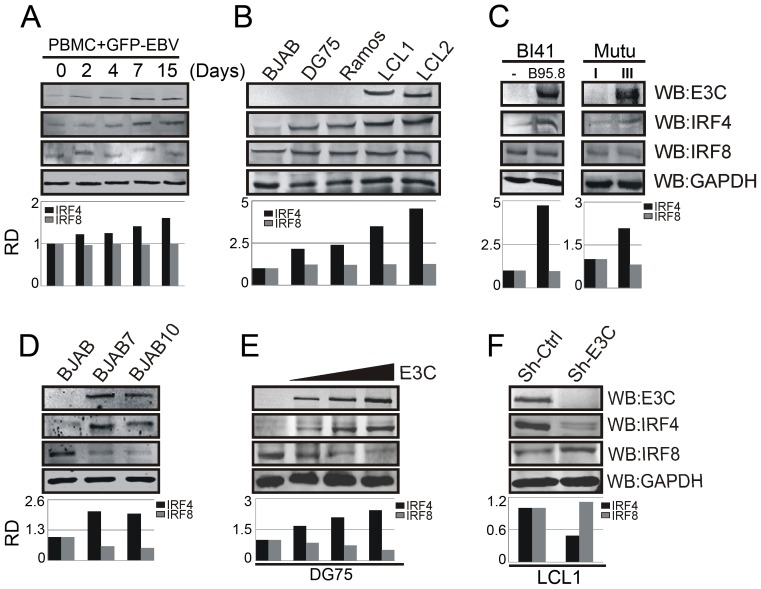
EBNA3C differentially regulates IRF4 and IRF8 expression. A) 10×10^6^ human PBMC (Peripheral blood mononuclear cells) were infected with BAC-GFP EBV for 4 hrs at 37°C. Cells were harvested after 0, 2, 4, 7, 15 days of post-infection and lysed in RIPA buffer. Western blot analysis was performed with indicated antibodies to detect specific endogenous proteins. B) 50 million EBV negative BJAB, DG75, Ramos and EBV transformed LCL1, LCL2 cells were harvested and total cell lysates were subjected to Western blot analysis (WB) using indicated antibodies. C) 20 million Burkitt's lymphoma (BL) cells BL41 and wild type EBV strain B95.8 infected BL41 cells, type I and III latency expressing BL cell lines-MutuI and MutuIII were lysed with RIPA buffer and Western blot analysis was performed with indicated antibodies. GAPDH was taken as internal loading control. D–F) 50 million D) BJAB, BJAB7, BJAB10 cells were harvested and Western blot analysis was performed using specific antibodies as indicated. E) EBV negative DG75 cells were transfected with increasing amount of EBNA3C expressing construct (0, 5, 10, 15 µg) and Western blot analysis was performed to detect IRF4, IRF8, EBNA3C, GAPDH proteins. F) Lentivirus mediated stable EBNA3C knockdown (Sh-E3C) or scramble control (Sh-Ctrl) LCL1 cells were subjected to Western blot analysis with indicated antibodies. Protein bands from Western blot analysis were analyzed by the Odyssey imager software and represented as bar diagrams based on internal loading control GAPDH.

To determine the status of IRF4 and IRF8 levels after EBV infection, 10 million human peripheral blood mononuclear cells (PBMC) were infected with BAC-GFP EBV and the cells were harvested at different time points. Western blot analysis showed up-regulation of IRF4 but no observed change in IRF8 protein level after 0, 2, 4, 7 and 15 days of post-infection (Fig. 1A). Western blot analysis of IRF4 and IRF8 levels were also performed in EBV transformed LCL1, LCL2 cells compared to EBV negative BJAB, DG75 and Ramos cell lines. The results showed a significant upregulation of IRF4 levels, but similarly IRF8 levels remained relatively unchanged (Fig. 1B). To further investigate this phenomenon, we analyzed the Burkitt's lymphoma cell line, EBV negative BL41 with and without the wild type EBV strain B95.8. Here again, BL41/B95.8 cells showed an increased level of IRF4 protein in this isogenic background which was not seen for IRF8. To determine if these differential protein expressions were related to expression of a specific EBV latent antigen expressed during latency type III, we analyzed the results from the Burkitt's lymphoma cell lines Mutu III (latency III) compared with Mutu I (latency I) [47]. Interestingly, a similar level of elevated IRF4 protein expression pattern was seen in Mutu III but again little or no detectable change seen in case of IRF8 (Fig. 1C).

To verify whether EBV infection was responsible for the change in *Irf4* compared to *Irf8* mRNA expression level as suggested by the above results, human peripheral blood mononuclear cells (PBMC) were infected with BAC-GFP EBV as previously described [48]. 0, 2, 4, 7 and 15 days post-infected samples were harvested for mRNA isolation and Real-time PCR analysis. Importantly, the result showed no detectable change in *Irf4* and *Irf8* mRNA levels (Supplementary Fig. S1A). Additionally, our real-time PCR analysis with EBV transformed LCL1 and LCL2 cells in comparison with EBV negative BJAB indicated no significant change in *Irf4* and *Irf8* mRNA expression levels (Supplementary Fig. S1B). To determine what effect EBNA3C may have on IRF4 and IRF8, EBV negative Burkitt's lymphoma cell line BJAB and BJAB7, BJAB10 (EBNA3C stably expressed in BJAB) cells were subjected to real-time PCR analysis. The data showed a similar mRNA expression pattern for *Irf4* and *Irf8* as before with no significant change (Supplementary Fig. S1C). Besides, we also compared the transcript level of EBNA3C in DG75, BJAB with LCL1, and LCL2. The result indicated that as expected the EBNA3C levels were substantially expressed in LCLs (Supplementary Fig. S1D). Therefore, these results confirm that regulation of IRF4 and IRF8 in EBV infected cells is most likely through post-translational modification of the polypeptides.

Previous reports showed that the EBV latent membrane protein 1 (LMP1) induced IRF4 expression [43]. To examine whether EBNA3C can directly regulate the expression of IRF4, we performed Western Blot analysis on P3HR1 and Jijoye cell lines, two isogenic Burkitt's Lymphoma lines [49]. Both cell lines are EBV positive and express EBNA3C, whereas P3HR1 lacks EBNA2 and a portion of EBNA-LP gene [50]. Consequently, due to that deletion, P3HR1 cells express negligible levels of LMP1 [51]. Our results clearly indicated that the expression levels of IRF4 are similar in these two cell lines (Supplementary Fig. S2). To explore the effect of EBNA3C on IRF4 and IRF8, EBV negative BJAB and BJAB7, BJAB10 cells were analyzed by Western blot. The results demonstrated a significant increase in IRF4 protein expression level and a substantial reduction of IRF8 expression (Fig. 1D). Therefore, these results further support a direct role for EBNA3C in regulation of IRF4 and IRF8 independent of other EBNAs or LMP1. We next investigated the protein expression levels of IRF4 and IRF8 with a dose-dependent increase of EBNA3C in EBV negative DG75 B-cell line. Our results showed that the IRF4 protein level steadily increased, but the detected IRF8 protein level decreased with increasing amounts of EBNA3C (Fig. 1E). To further elucidate the role of EBNA3C in modulating the levels of IRF4 and IRF8, we performed Western blot analyses on LCLs which were stably knocked-down for EBNA3C with specific EBNA3C short hairpin RNA (Sh-E3C). We observed that the IRF4 protein expression levels were significantly decreased as compared to the sh-control (Sh-Ctrl) LCLs. However, the protein expression level of IRF8 was found higher in EBNA3C knocked-down cells, when compared with the sh-control vector stably transfected LCL1 (Fig. 1F).

### IRF4 and IRF8 form complexes with EBNA3C in EBV-transformed LCLs

**Figure 2:**
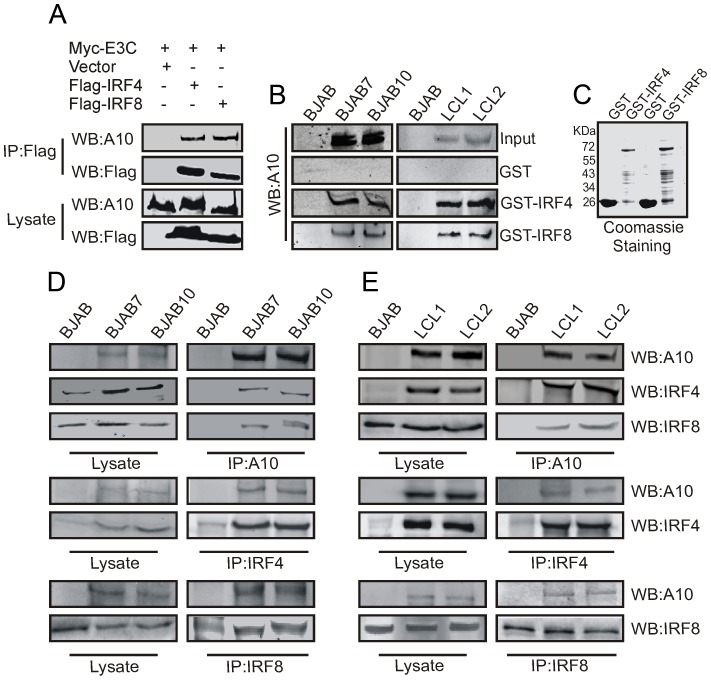
EBNA3C physically associates with IRF4 and IRF8. A) 10 million HEK- 293 cells were co-transfected with Myc-tagged EBNA3C and Flag-tagged IRF4, IRF8 vectors. Control samples were balanced by using empty vector. Transfected cells were harvested at 36 hrs of post-transfection and approximately 5% of the lysates were used as input and the residual lysate was immunoprecipitated (IP) with 1 µg of anti-Flag (M2) antibody. Lysates and Immunoprecipitated samples were resolved by 8% SDS-PAGE and western blot (WB) analysis was performed with the indicated antibodies. B) 50 million EBV negative BJAB, BJAB cells stably expressing EBNA3C (BJAB7, BJAB10) and two different clones of EBV transformed lymphoblastoid cell lines as- LCL1, LCL2 cells were harvested and lysed in RIPA buffer. Cell lysates were incubated with either GST control or GST-IRF4 or GST-IRF8 beads. EBNA3C protein was detected by western blot analysis using EBNA3C specific monoclonal antibody (A10). C) Purified control GST and GST-IRF4, GST-IRF8 proteins used in this experiment were resolved by 10% SDS-PAGE and stained with Coomassie Blue. 50 million D–E) BJAB, BJAB7, BJAB10, LCL1, LCL2 cells were lysed and immunoprecipitation was performed by A10, IRF4, IRF8 specific antibodies. Immunoprecipitated samples were resolved by 8% SDS-PAGE and endogenous EBNA3C, IRF4, IRF8 proteins were detected by their specific antibodies.

We further examined whether IRF4 and IRF8 can directly associate with EBNA3C. First, we performed in vivo co-immunoprecipitation assays in EBV positive cells. In addition, HEK-293 cells were co-transfected with expression constructs for Myc-tagged EBNA3C and IRF4, IRF8 tagged with the Flag epitope. Immunoprecipitation analysis as shown in Fig. 2A, clearly demonstrated a strong association of EBNA3C with IRF4 and IRF8 in cells. Further, we checked the interactions using GST pull-down experiments, with cell lysates prepared from two transformed EBV-positive lymphoblastoid cell lines-LCL1 and LCL2, EBV negative BJAB and EBNA3C expressing BJAB7 and BJAB10. The results demonstrated that EBNA3C strongly interacted with the GST-IRF4 and GST-IRF8 fusion proteins but not with the GST control (Fig. 2B). The amount of control GST and GST-IRF4, GST-IRF8 fusion proteins used in this GST-pulldown experiment was shown in a parallel gel with Coomassie Blue staining (Fig. 2C). To further support this association as on endogenous complex, EBNA3C, IRF4 or IRF8 were immunoprecipitated from BJAB, EBV transformed LCL1 and LCL2 cells, as well as BJAB7 and BJAB10 (Fig. 2D, E). Data analyzed from the ectopic expression as well as LCLs endogenously expressing IRF4, IRF8 and EBNA3C confirmed a substantial association of IRF4 and IRF8 with EBNA3C in a molecular complex in EBV-infected cells.

### A small N-terminal domain of EBNA3C interacts with IRF4 and IRF8

**Figure 3:**
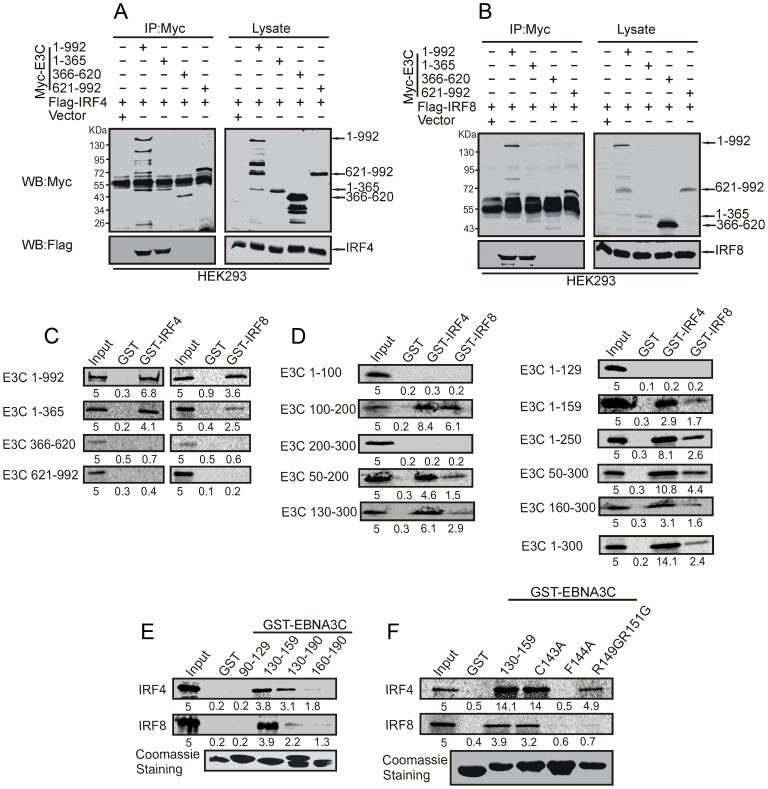
The N-terminal domain of EBNA3C interacts with IRF4 and IRF8. A) 10 million HEK-293 cells were transfected with either control vector or Full length and different truncated mutants of Myc-tagged EBNA3C with A) Flag-tagged IRF4 or, B) Flag-tagged IRF8 plasmid constructs. After 36 hours of post-transfection, cells were harvested and immunoprecipitation performed with 1?g of anti-Myc antibody. IP samples were resolved in 10% SDS-PAGE. Western blot was performed with anti-Myc and anti-Flag antibody. C) Full length and different domains truncated mutant constructs of EBNA3C (residues 1-992, 1-365, 366-620 and 621-992) were in vitro translated using a T7-TNT translation kit. After pre-clearing with GST-beads, all S35-radiolabeled in vitro translated proteins incubated with either GST control or GST-IRF4, GST-IRF8 beads. Reaction samples were washed with Binding Buffer and resolved by 10% SDS-PAGE, exposed to phosphoimager plate and scanned by Typhoon Scanner. D) A series of different N-terminal truncated mutants of EBNA3C were used for in vitro translated S35-radiolabeled protein production and their binding affinity determined with GST-IRF4 or GST-IRF8 as similar to C). E) Flag-IRF4 and IRF8 constructs were used for in vitro translation and S35-radiolabeled in vitro translated proteins were incubated with either GST control or different GST-EBNA3C truncated mutants specific for residues 90-129, 130-159,130-190, 160-190 beads. Coomassie staining of SDS-PAGE resolved purified GST proteins is shown in the bottom panel of E). F) Wild type GST-EBNA3C (residues 130-159) and different GST-mutant EBNA3C were expressed in E. coli and purified with Glutathione Sepharose beads.Flag-IRF4 and IRF8 plasmid constructs were used for in vitro translation and S35-radiolabeled in vitro translated proteins were incubated with either GST control or Wild type GST-EBNA3C (residues 130-159) and different GST-mutant EBNA3C. Coomassie staining of SDS-PAGE resolved purified control, wild type and mutant GST proteins are shown in the bottom panel.In each case, 5% of IVT input was used for the comparison. Relative binding units (RBU) were indicated as numerical values at the bottom of IVT gel.

In order to determine the specific domain of EBNA3C that interacts with IRF4 and IRF8, HEK-293 cells were co-transfected with expression constructs for Flag-IRF4, Flag-IRF8, Myc-tagged full length EBNA3C (residues 1–992) or different truncated mutant of EBNA3C (residues 1–365, 366–620 or 621–992) and co-immunoprecipitation experiment was performed with anti-Myc antibody. The result illustrated that only the full length or N-terminal truncated EBNA3C co-immunoprecipitates with IRF4 and IRF8. On the other hand, no co-immunoprecipitation was observed with other truncated mutants of EBNA3C (Fig. 3A, B). To further corroborate the association results, we performed in-vitro GST-pulldown assays using in-vitro translated full length and truncated EBNA3C mutant fragments followed by the co-incubation with bacterially expressed and purified GST-IRF4 and GST-IRF8 proteins. The result indicated that the N-terminal EBNA3C directly interacted with both IRF4 and IRF8 (Fig. 3C). Next, we extended our in-vitro GST-pulldown experiment to determine the specific residues within the N-terminal domain of EBNA3C, important for the IRF4 and IRF8 interactions. We further found with in-vitro precipitation assays using a series of N-terminal truncated mutants of EBNA3C that the N-terminal amino acid residues 130–190 bound to IRF4 and IRF8 (Supplementary Fig. S3A), however, amino acid 130–159 residues showed the highest binding affinity (Fig. 3D, E). To determine the importance of specific amino acid residues mediating these interactions, we observed some functionally conserved residues from an alignment result of EBNA3C 130–159 with EBNA3A and EBNA3B (Supplementary Fig. S3B). To address the critical role of individual amino acid residues, single or double point mutation were introduced in conserved EBNA3C 130–159 residues as indicated by boxes in Fig. 3G. Interestingly, Phenylalanine 144 (F144A) dramatically reduced the interaction with IRF4 and IRF8. In addition, a double mutation of arginine-149 and arginine-151 also significantly reduced IRF4 and IRF8 binding activity (Fig. 3F).

### The C-terminal domain of IRF4 and IRF8 physically interacts with EBNA3C and the Spi-1/B-like motif of IRF4 is important for this interaction

**Figure 4:**
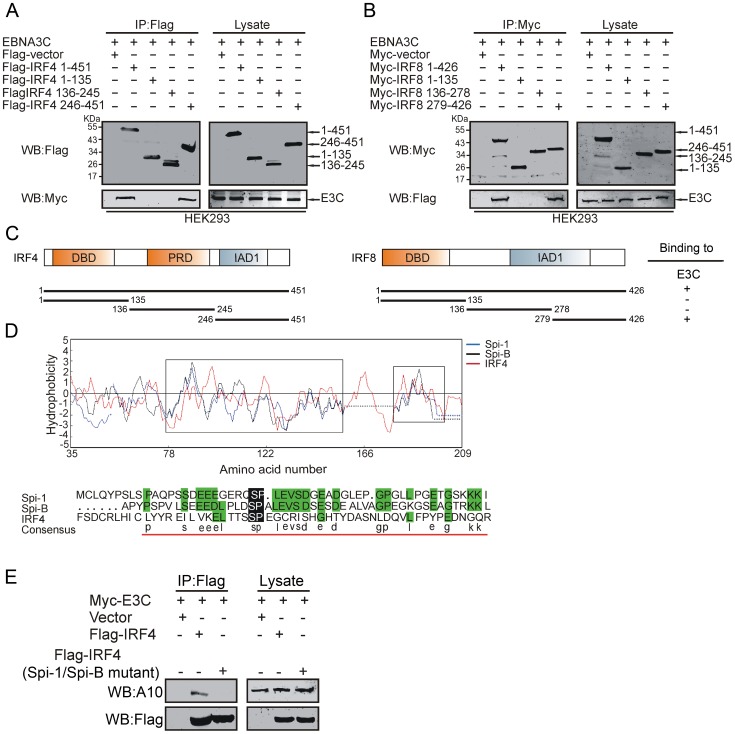
The C-terminal domain of IRF4 and IRF8 binds with EBNA3C and Spi-1/B-like motif is important for IRF4 interaction. A) 10 million HEK-293 cells were transfected with either control vector or different truncated mutants of Flag-tagged IRF4 with Myc-EBNA3C and B) Myc-tagged IRF8 truncated mutants with Flag-tagged EBNA3C. After 36 hours of post transfection, cells were harvested and immunoprecipitation was performed with 1 µg of anti-Flag or anti-Myc antibodies. IP samples were resolved in 10% SDS-PAGE. Western blot was performed with anti-Myc and anti-Flag antibody. C) The schematic represents different structural and interactive domains of IRF4 and IRF8. Binding affinities of different domains of IRF4 and IRF8 with EBNA3C was also summarized. +, binding; −, no binding. D) Hydrophobicity graph of Spi-1, Spi-B and IRF4 sequence alignment (upper panel). Spi-1, Spi-B and IRF4 protein sequences alignment (lower panel). Spi-1/B-like consensus sequence is underlined and based on the hydrophobic homology. E) 10 million HEK-293 cells were transfected with either control vector or Myc-tagged EBNA3C with either wild type or Spi-1/B motif deleted Flag-IRF4 plasmid constructs. IP was performed with 1 µg anti-Flag antibody. Western blot was performed with anti-Myc and anti-Flag antibodies.

To define the specific domain of IRF4 and IRF8 responsible for interacting with EBNA3C, we performed co-immunoprecipitation experiments using Flag-tagged full length (resides 1–451) and different truncated mutants of IRF4 (residues 1–135, 136–245, 246–451) or IRF8 (residues 1–426, 1–135, 136–278, 279–426) with Myc-tagged full length EBNA3C. The results indicated that the C-terminal domain of IRF4 and IRF8 bound to EBNA3C (Fig. 4A, B, C). Previously, it was reported that the amino terminal domain of EBNA3C associates with Spi-1 and Spi-B proteins [52]. It is also known that IRF4 directly associates with Spi-1 and Spi-B [53], [54]. So, it was possible that IRF4 interacts with EBNA3C through a Spi-1 or Spi-B motif. To further examine if IRF4 interacts with EBNA3C through its Spi-1/B motif, we first aligned the sequence of Spi-1, Spi-B and IRF4 proteins. Alignment data analysis showed that IRF4 contains a Spi-1/B motif based on hydrophobic homology of amino acid sequences and that the consensus Spi-1/B motif lies within residues 250–295 (Fig. 4D). To further confirm the association of IRF4 and EBNA3C through Spi-1/B motif, co-immunoprecipitation assays were performed with Myc-tagged full length EBNA3C, full length Flag-IRF4 and Flag-Spi-1/B deletion mutant of IRF4. The result clearly demonstrated that the interaction with EBNA3C and IRF4 is significantly less with the Spi-1/B motif deletion mutant suggesting that this motif is important for its interaction (Fig. 4E).

### EBNA3C co-localizes with IRF4 and IRF8 in nuclear compartments

**Figure 5:**
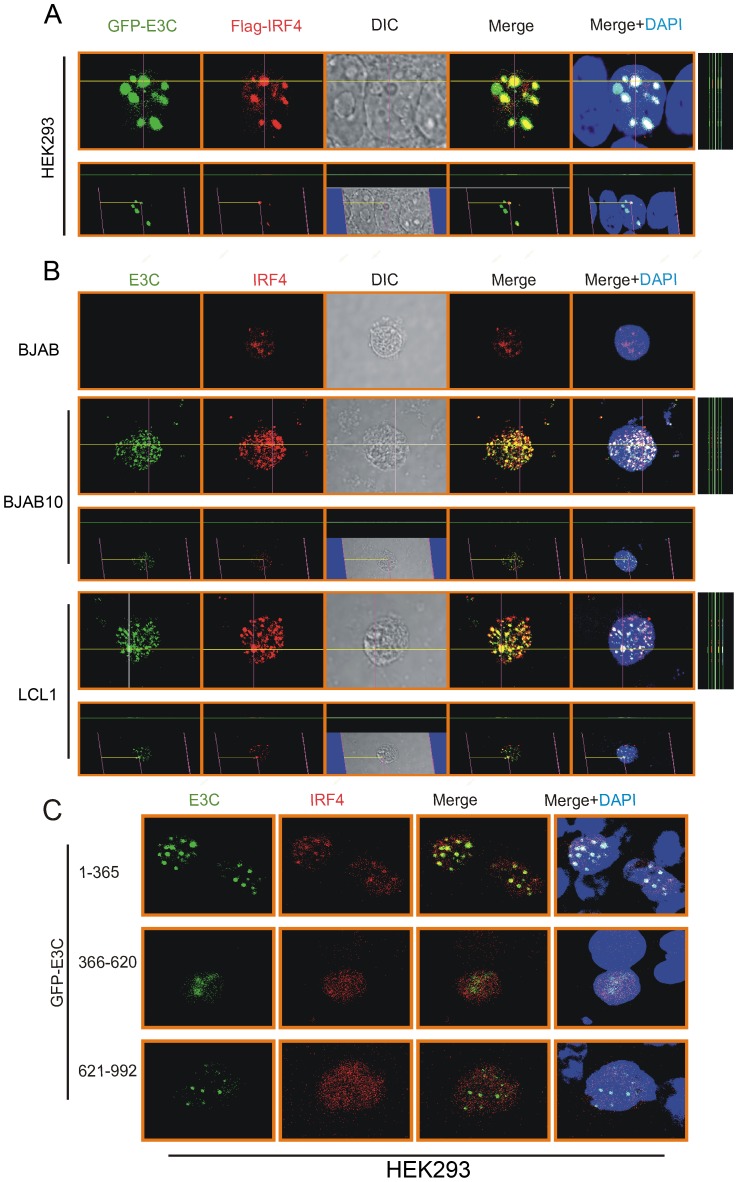
IRF4 co-localizes with EBNA3C in human cells. A) 0.3 million HEK-293 cells plated on coverslips and transiently transfected with Control vector, GFP-EBNA3C and Flag-IRF4 expression vectors by using Lipofectamine 2000 transfection reagent. B) BJAB, BJAB10, LCL1 cells were plated on slides and air-dried. C) 0.3 million HEK-293 cells were grown on coverslips and transiently transfected with different truncated domains of GFP-EBNA3C (residues 1–365, 366–620, 621–992) and Flag-IRF4 expression vectors by using Lipofectamine 2000 transfection reagent. Cells were fixed using 3% PFA. Ectopic and endogenous expressions of IRF4 was detected using anti-Flag (M2)-antibody (1∶200 dilution) and IRF4 specific antibody (1∶50 dilution) respectively, followed by anti-Rabbit Alexa Fluor 594 (red) as secondary antibody. Endogenous EBNA3C was detected using A10 ascites (1∶150 dilution) followed by anti-mouse Alexa Fluor 488 (green). DAPI (49, 69-diamidino-2-phenylindole) was used (1∶500 dilution) to stain nuclei. The images were captured by Olympus Fluoview confocal microscope.

The above studies showed a complex with EBNA3C, IRF4 and IRF8. Furthermore, previous experiments suggested that there was a dramatic upregulation of IRF4 in the presence of EBNA3C. Therefore, we examined the sub-cellular localization of IRF4 and EBNA3C. Immunofluorescence analysis was performed in transiently transfected as well as EBV transformed cells. We ectopically expressed Flag-tagged IRF4 with the GFP-tagged EBNA3C in HEK-293 cells. The results clearly demonstrated that IRF4 co-localized with EBNA3C in nuclear compartments with the exclusion of the nucleoli as indicated by yellow fluorescence signals (Fig. 5A). To further validate our finding and to also support the association between IRF4 and EBNA3C under relevant physiological conditions, we used BJAB10 stably expressing EBNA3C and LCL1 cells. Here, BJAB cells represent EBV negative cells which do not express EBNA3C. Using specific antibodies against EBNA3C and IRF4, our immunofluorescence studies clearly indicated that EBNA3C signals were predominantly co-localized with IRF4 to similar nuclear compartments. (Fig. 5B). A similar result was obtained with IRF8 although the signals were much weaker and is likely due to the reduced levels of IRF8 (data not shown). Interestingly, some IRF4 signals were also diffused in the nucleus although a large portion of those clearly co-localized with the punctate signals of EBNA3C (Fig. 5A and 5B). Additionally, the co-localization study was extended with different truncated regions of EBNA3C and the result shown here, demonstrated that IRF4 co-localized with the N-terminal domain of EBNA3C (residues 1–365) whereas, the middle domain (residues 366–620) and C-terminal domain (residues 621–992) showed negligible co-localization pattern (Fig. 5C).

### EBNA3C is critical for enhanced stabilization of IRF4

**Figure 6:**
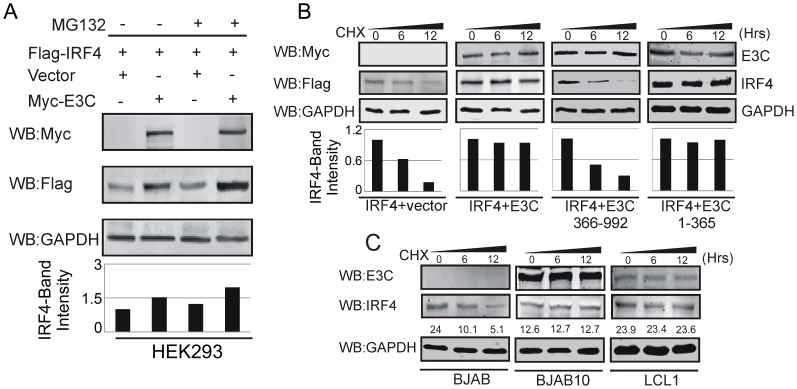
EBNA3C stabilizes IRF4. A) 10 million HEK-293 cells were co-transfected with Flag-IRF4 and either vector control (lanes 1 and 3) or Myc-EBNA3C (lanes 2 and 4) expression constructs. After 36 hrs of post-transfection, transfected cells were treated with either 40 µM MG132 (+ lanes) or DMSO (- lanes) for additional 6 hrs and cell lysates were resolved by 8% SDS-PAGE and Western blot was performed with the indicated antibodies. B) HEK-293 cells were transfected with Flag-tagged IRF4 and vector control, full length, N-terminal domain expressing or N-terminal domain deleted mutant Myc-tagged EBNA3C plasmid vectors. At 36 hrs of post-transfection, cells were treated with 40 µg/ml cyclohexamide (CHX) for 0, 6, 12 hrs. Cells were lysed and protein samples were resolved by 8% SDS-PAGE. Western blot was performed by specific antibodies shown. C) BJAB, BJAB10, LCL1 cells were treated with 40 µg/ml cyclohexamide (CHX) for indicated time periods. Cell lysates were resolved by 8% SDS-PAGE. Western blot analysis was performed with indicated antibodies. GAPDH blot was shown for internal loading control. Protein bands quantitation was performed by Odyssey imager software as arbitrary numerical values indicated at the bottom of gel.

From the studies above it was clear that EBNA3C can play a major role or is a major contributor to IRF4 protein levels. To determine whether EBNA3C can modulate the stabilization of IRF4, we co-transfected HEK-293 cells with Flag-tagged IRF4, and either the control vector or the Myc-tagged EBNA3C and then treated the transfected cells with the proteasome inhibitor MG132. Interestingly, the results indicated a significant accumulation of IRF4 protein in comparison with mock treated or control vector transfected cells (Fig. 6A). Next, HEK-293 cells were transfected with control vector, Flag-IRF4, full length, N-terminal (residues 1–365) and N-terminal deleted mutant (residues 366–992) of Myc-tagged EBNA3C expression constructs. Thirty-six hours post-transfected cells were treated with the protein synthesis inhibitor, cyclohexamide and monitored for protein levels at 0, 6 and 12 hours by Western blot analysis. As expected, the results demonstrated that the stability of IRF4 protein was significantly increased in the presence of EBNA3C (Fig. 6B). However, in the absence of EBNA3C or using the specific N-terminal deleted EBNA3C, the IRF4 protein levels were gradually degraded within 6 to 12 hours of cyclohexamide treatment (Fig. 6B). This result therefore provided evidence as to the stability of both EBNA3C and IRF4 proteins when the protein synthesis inhibitor cyclohexamide was used in the assay and also demonstrated a critical role for N-terminal domain of EBNA3C in enhancing IRF4 stability (Fig. 6B). We also performed a similar protein stability assay for BJAB, BJAB10 and LCL1 cells treated with cyclohexamide. Our results reconfirmed that in the EBNA3C expressing BJAB10 cell line, as well as in LCLs expressing other latent proteins the IRF4 levels were greatly enhanced (Fig. 6C). Therefore, EBNA3C contributes to stabilization of IRF4 in EBV infected and transformed cells.

### EBNA3C stabilizes IRF4 but not IRF8 through regulation of polyubiquitination

**Figure 7:**
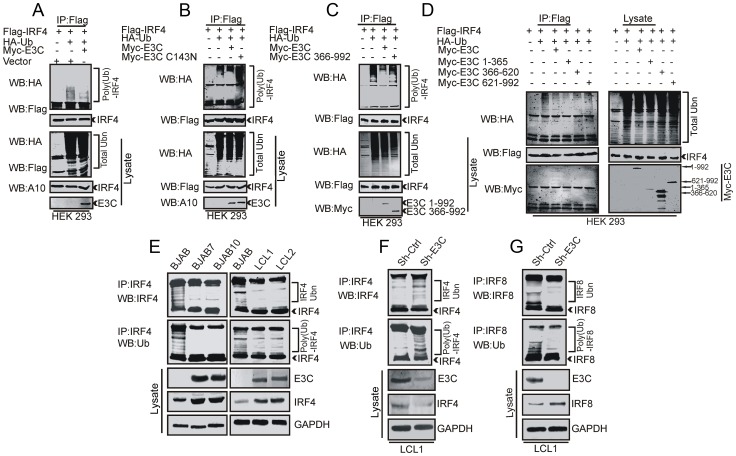
EBNA3C differentially modulates poly-ubiquitination of IRF4 and IRF8. A, B, C, D) 10 million HEK-293 cells were transiently transfected with different combinations of expression vectors as indicated. Cells were harvested after 36 hrs of post-transfection and total protein was immunoprecipitated (IP) with indicated antibodies and protein samples were resolved by 10% SDS-PAGE. Western blots were performed by stripping and re-probing the same membrane. E) 50 million EBV negative BJAB cells, BJAB cells stably expressing EBNA3C (BJAB7, BJAB10) and EBV transformed LCL1, LCL2 were incubated with proteasome inhibitor MG132 drug (20 µM) for 6 hrs. Cells were harvested and lysed with RIPA buffer. IRF4 was immunoprecipitated (IP) by using specific antibodies. Samples were resolved by 10% SDS-PAGE. Western blotting (WB) was performed by stripping and reprobing the same membrane. F–G) Ubiquitination assay was performed with Lentivirus mediated stable EBNA3C knockdown (Sh-E3C) or scramble control (Sh-Ctrl) LCL1 cells and subjected to Western blot analysis with indicated antibodies.

Our previous studies showed that EBNA3C targets the SCF^Skp2^ E3 ubiquitin (Ub) ligase complex thereby destabilizing various key cell-cycle proteins including retinoblastoma (Rb) and p27 KIP [55]. Recent experimental evidence also suggested that EBNA3C can enhance the intrinsic ubiquitin ligase activity of Mdm2 towards P53, which in turn aided degradation of p53 by ubiquitination [56]. Thus, by modulating cellular ubiquitination activities, EBNA3C can provide a favourable environment for malignant transformation and proliferation of EBV-infected cells. The above-mentioned results demonstrated stabilization of IRF4 particularly in the presence of EBNA3C. We further assessed the possible contribution of EBNA3C to modulation of the poly-ubiquitination status of IRF4 and IRF8. To further corroborate our hypothesis, we performed ubiquitination assays using HEK-293 cells. Here, we transiently co-transfected different expression constructs for HA-Ub, Flag-IRF4 and Myc-EBNA3C. Furthermore, immunoprecipitation followed by Western blot analysis clearly showed a reduction in the level of IRF4 poly-ubiquitination in EBNA3C expressing cells (Fig. 7A). Next, we extended this experiment using the catalytic domain mutant of EBNA3C Myc-C143N and N-terminal deleted mutant Myc-EBNA3C (residues 366–992). The results showed enhanced ubiquitination of IRF4 when transfected with these expressions constructs (Fig. 7B, C). Additionally, we performed ubiquitination assays with different truncated mutants of EBNA3C in HEK-293 cells. The results also confirmed a critical role for the N-terminal domain of EBNA3C in regulation of IRF4 poly-ubiquitination (Fig. 7D). Additionally, we performed immuno-precipitation assays using specific antibodies against IRF4 in EBV negative BJAB, and BJAB7, BJAB10 cells, as well as EBV transformed LCL1, LCL2 cells. The complexes showed high molecular weight species of IRF4 (Fig. 7D). These high molecular weight species migrated at a slower rate in EBV-negative BJAB cells. However, in BJAB7 and BJAB10 or in LCL1 and LCL2 there were significantly less compared to the EBV negative BJAB cells. Western blot analysis using Ubiquitin-specific antibody showed a similar pattern (Fig. 7E). We also evaluated IRF4 ubiquitination using a sh-control vector and a Sh-E3C stably transfected LCL1 cell lines. The resulting poly-ubiquitination of IRF4 was shown to be significantly higher in EBNA3C knockdown. However, the poly-ubiquitinated status of IRF8 was found to be much reduced in the same cell lines (Fig. 7F and 7G). These results strongly indicate that EBNA3C differentially modulates ubiquitination of IRF4 and IRF8, and so their stability in EBV transformed cells.

### EBNA3C is important for IRF4-mediated regulation of IRF8 and its target proteins

**Figure 8:**
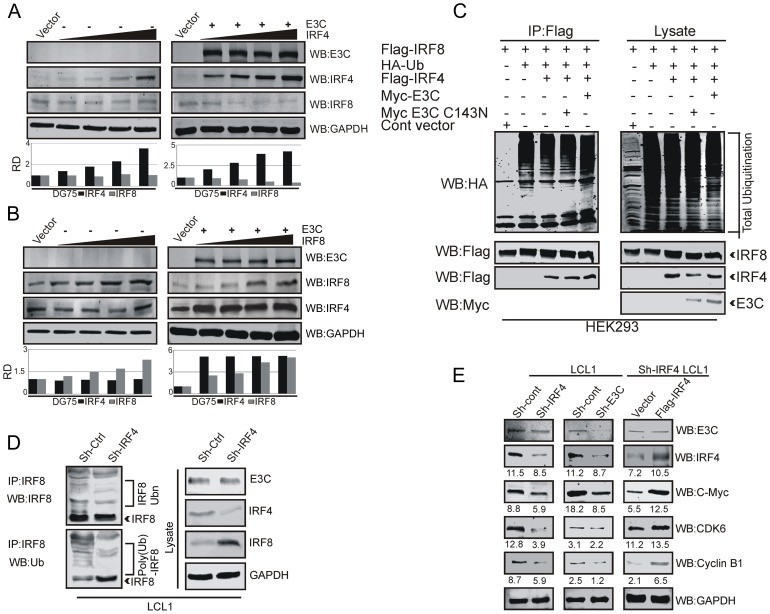
EBNA3C is important for IRF4-mediated downregulation of IRF8 and activation of IRF4 target proteins. A–B) 50 million EBV negative DG75 cells were transfected with increasing amount of A) IRF4 and B) IRF8 expression vector (0, 5, 10, 15 µg) without EBNA3C (left panel) or with 10 µg EBNA3C (right panel). After 36 hrs of post-transfection, cells were harvested and lysed with RIPA buffer. Protein samples were subjected to Western blot analysis using EBNA3C, IRF4, IRF8 specific antibodies. GAPDH western blot was performed for loading control. C) 10 million HEK-293 cells were transiently transfected with different combinations of expression vectors as indicated. After 36 hrs of post-transfection, cells were treated with MG132 for additional 6 hrs and total protein was immunoprecipitated (IP) with indicated antibodies. Protein samples were resolved by 10% SDS-PAGE. Western blots were performed by stripping and re-probing the same membrane. D) 50 million Sh-Ctrl, Sh-IRF4 LCL1 cells were incubated with proteasome inhibitor MG132 (20 µM) for 6 hrs. Cells were harvested and lysed with RIPA buffer. IRF8 protein was immunoprecipitated (IP) by using IRF8 specific antibody. Immunoprecipitated samples were resolved by 10% SDS-PAGE. Western blot analysis was performed by using specific antibodies shown. E) 50 million stable Sh-Ctrl, Sh-IRF4, Sh-E3C LCL1 and Ctrl-vector and Flag-IRF4 transfected, Sh-IRF4 LCL1 cells were lysed in RIPA buffer and Western blot was performed to show the expression levels of EBNA3C, IRF4, c-Myc, Cyclin-dependent kinase 6, Cyclin B1 and GAPDH.

To explore the potential mechanism of EBNA3C-mediated IRF4 induction and degradation of IRF8, we transiently transfected EBV negative DG75 cell line with control vector, with or without EBNA3C tagged with the Myc epitope (Myc-EBNA3C), and an increasing dose of IRF4 tagged with the Flag epitope (Flag-IRF4). We monitored the expression of IRF8 protein by Western Blot analysis. Surprisingly, we observed no significant change of IRF8 protein levels in EBNA3C untransfected control, but a significant down-regulation of IRF8 was seen in the presence of EBNA3C (Fig. 8A). We then performed Western blot analysis by transfecting DG75 cells with or without Myc-EBNA3C and an increasing dose of Flag-IRF8 to monitor IRF4 protein levels. Our results demonstrated that even with the gradual increase of IRF8 expression, the IRF4 protein expression level was relatively unchanged as determined by Western blot in the presence or absence of EBNA3C (Fig. 8B). To investigate whether IRF4 is playing a vital role in IRF8 degradation in the presence of EBNA3C, we performed poly-ubiquitination assays by co-transfecting Flag-IRF4, IRF8, Myc-tagged EBNA3C and the Myc-tagged catalytic domain mutant of EBNA3C in HEK-293 cells. Interestingly, in the presence of wild-type EBNA3C we found higher ubiquitination of IRF8 with IRF4 as compared to EBNA3C-C143N (Fig. 8C). These results further corroborated the above data showing the negative regulation of IRF8 by IRF4 in the presence of EBNA3C. To explore the effect of the modulation of IRF4 on IRF8 protein levels in the context of EBV-mediated B-cell transformation, we stably transduced LCL1 cells with lentivirus which express a short hairpin RNA to knock-down IRF4 (Sh-IRF4). In parallel, we used stable Sh-Ctrl LCL1 cells as a control cell line. We also verified that the cells were stably transduced by checking the GFP expression and the protein expression level of IRF4 knock-down cells by Western blot analysis (Supplementary Fig. S4A, B). Previously, we observed a decrease in poly-ubiquitination of IRF8 in EBNA3C knockdown LCL1 cells (Fig. 7F), whereas there was enhanced ubiquitination of IRF4 as seen by an increased pattern of the slower migratory bands. Here, we further investigated the polyubiquitination status of IRF8 in stable IRF4 knockdown LCL1 cells compared with Sh-Ctrl LCL1 cells. Surprisingly, we found a reduction in the IRF8 poly-ubiquitination level and also a substantial upregulation of IRF8 upon IRF4 knockdown (Fig. 8D). This strongly suggests that IRF4 is a major player in EBNA3C-mediated IRF8 destabilization and degradation by the Ubiquitin-proteasome system.

Previous studies suggested that the oncogenic transcription factor c-Myc has a crucial role in cell growth, metabolism and proliferation [57], Cyclin dependent kinase 6 (CDK6) overexpression was implicated in the pathogenesis of B-cell malignancies [58], and also aberrant expression of the cell cycle regulatory protein Cyclin B1 has been detected in different lymphomas and EBV-immortalized cells [59], [60]. Earlier studies also demonstrated that these molecules are direct targets of IRF4 [44], [61]. To determine, if EBNA3C has some regulatory effect on the IRF4 downstream signaling molecules, we checked the status of the protein levels of these molecules in IRF4 and EBNA3C stable knockdown LCL1 cells. The results showed down regulation of those proteins upon IRF4 or EBNA3C knockdown (Fig. 8E). To validate this data, the IRF4 stable knockdown LCL1 cells were transiently transfected with either control vector or Flag-tagged IRF4 plasmid vectors. 48 hrs after transfections the cells were harvested and Western blot analysis was performed (Fig. 8E). The results clearly indicated that rescue of IRF4 expression in IRF4 stable knockdown LCL1 cells had similar results compared to wild-type for expression levels of c-Myc, Cyclin-dependent kinase 6 and Cyclin B1 which were substantially increased compared to the control vector cells. Dysregulation of these downstream signaling molecules may play a major role in a range of cellular process, such as aberrant cell proliferation, apoptosis and cell-cycle regulation.

### EBNA3C is crucial for IRF4 mediated cellular proliferation by inhibiting apoptosis

**Figure 9:**
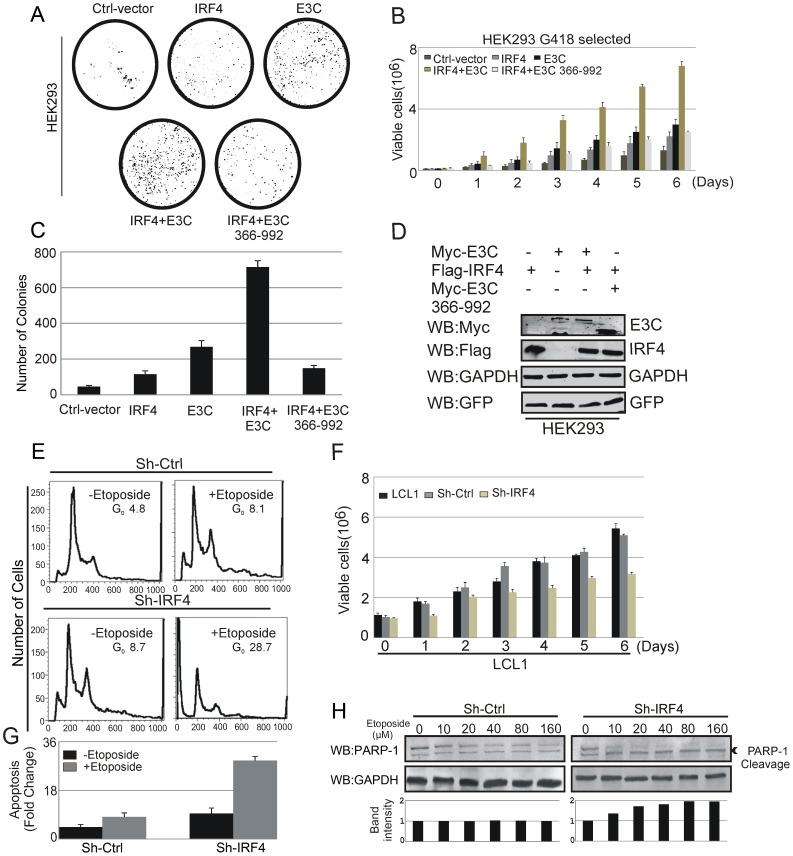
EBNA3C is critical for IRF4-mediated cell proliferation by inhibiting apoptosis. 10 million HEK-293 were transfected with Ctrl-vector and different combinations of expression plasmids for Myc-tagged EBNA3C, Flag-tagged IRF4 and N-terminal domain deleted mutant Myc-tagged EBNA3C (residues 366–992). In addition, cells were transfected with GFP expression vector. Transfected cells were selected for 2 weeks with G418 antibiotic. A) After 2 weeks of selection, GFP fluorescence of each plate was scanned by a PhosphorImager and the area of the colonies calculated by Image J software. C) The numbers of colonies in different transfected sets are represented in bar diagram. The data represented in bar diagram is the average of three independent experiments. B) 0.1×10^6^ cells from each set of selected samples were plated and cultured for 6 days. Viable cells were counted at indicated time points by trypan blue dye exclusion technique. D) G418 selected stable cells were harvested, lysed in RIPA buffer and subjected to immunoblot analyses with indicated antibodies. E, F, H) Sh-Ctrl, Sh-IRF4 LCL1 cells were grown in RPMI medium for 12 hrs with or without etoposide. E) Cells were harvested and stained with Propidium iodide. Stained cells were subjected to Flowcytometric analysis. G) The bar diagram represents the fold change of apoptosis seen by cell cycle analysis using FACS. The results shown are representative of three independent experiments. F) 1×10^6^ cells were plated and allowed them to grow at 37°C in complete medium without puromycin antibiotic. Viable cells were counted by trypan blue dye exclusion technique at indicated time points. The results shown here are representative of three independent experiments. H) Cells were treated with increasing amount (0, 10, 20, 40, 80, 160 µM) of etoposide drug for 12 hrs. Cell lysates were used for Western blot analysis with PARP-1, GAPDH antibodies.

In order to examine the effect of EBNA3C on IRF4 mediated cell proliferation, HEK-293 cells were transfected with control vector, Myc-tagged EBNA3C, Flag-tagged IRF4 expression plasmids, Myc-tagged EBNA3C and Flag-tagged IRF4, Myc-tagged N-terminal domain deleted mutant EBNA3C (residues 366–992) and Flag-IRF4 as designated in the figure (Fig. 9A–C). In addition, cells were also transfected with GFP expression vector. Transfected cells were selected with G418 for 2 weeks for colony formation assays. Here, we observed a significant increase in colony numbers when EBNA3C and IRF4 proteins were co-transfected in comparison to the set transfected with only IRF4. Interestingly, co-expression with the N-terminal domain deleted mutant of EBNA3C and IRF4 showed a significant reduction in colony numbers which also confirmed the importance of N-terminal EBNA3C domain in IRF4 mediated cellular proliferation (Fig. 9A and 9C). We further extended these studies by performing cell proliferation assays as determined by cell counting for 6 days (Fig. 9B). By using Trypan Blue dye staining, dead cells were excluded. To determine the expression levels of the transfected constructs, Western blot analysis was performed with G418 selected stable cells (Fig. 9D).

Recently, it was reported that inhibition of IRF4 is deleterious to myeloma cell lines suggesting that IRF4 silencing can be a potentially new target to be exploited therapeutically [62]. To corroborate these studies that IRF4 has oncogenic potential and can prevent cellular apoptosis, we performed Flow-cytometric analysis of etoposide treated Sh-Ctrl, Sh-IRF4 LCL1 cells to examine the cell-cycle distribution profiles. Our result showed an increased percentage of sub-G_0_/G_1_ phase cells in IRF4 knockdown cells with etoposide treatments (Fig. 9E). Moreover, the results showed that IRF4 knockdown LCLs were more prone to chemo-therapeutic drug induced cell death and clearly demonstrated that the level of apoptosis was much higher for Sh-IRF4 LCL1 cells compared to Sh-Ctrl LCL1 cells (Fig. 9G). To assess the cellular proliferation in context of IRF4 knock-down, a proliferation assay was performed at different time points by treating cells with etoposide. Interestingly, the result indicated that the proliferation rate of IRF4 knock-down LCL1 cells was lower compared to the Sh-Ctrl LCL1 (Fig. 9F). We also performed cell proliferation assays with two different EBV negative cell lines DG75 and BJAB, EBV transformed cell lines LCL1 and LCL2, and EBNA3C expressing BJAB7 and BJAB10 cells, along with Sh-Ctrl and Sh-E3C transfected stable LCL1 cell lines. These results clearly suggested that EBV positive cells specifically expressing EBNA3C were not susceptible to etoposide induced cell death. However, in EBNA3C knockdown cells, significant reduction of cell proliferation was observed (Supplementary Fig. S5). To further confirm whether IRF4 silencing had some effect on etoposide induced apoptosis of EBV positive lymphoblastoid cells, Western blot analysis was performed to examine the level of Poly (ADP-ribose) polymerase 1 (PARP-1) cleavage. Sh-Ctrl and Sh-IRF4 LCL1 cells were treated with different concentrations of etoposide and were examined to detect the 89 kDa active form of PARP-1 by Western blot analysis. The results clearly demonstrated that EBV transformed cells with IRF4 knock-down showed an increased sensitivity to etoposide induced apoptosis (Fig. 9H). Additionally, we performed an assay to detect apoptotic cells with ethidium bromide and acridine orange staining as well as DNA fragmentation assay. The results also indicated that IRF4 knockdown enhanced the level of apoptosis in EBV transformed LCL1 cells (Supplementary Fig. S6 and S7).

### EBNA3C enhances the oncogenic potential of IRF4 and attenuates IRF8 induced apoptosis to promote cell proliferation

**Figure 10:**
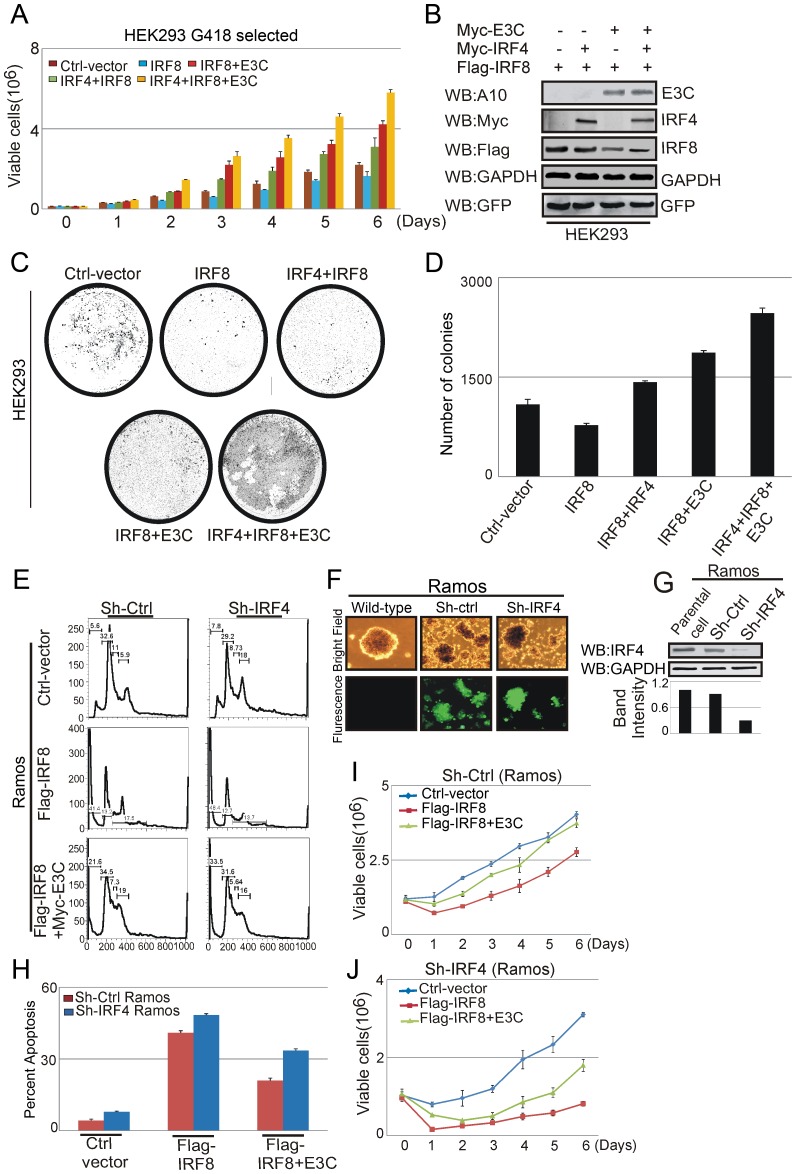
EBNA3C promotes IRF4-mediated cell proliferation by inhibiting IRF8 induced apoptosis. A–D) Human Kidney embryonic cells (HEK-293) were transfected with Ctrl-vector and different combinations of expression plasmids for Myc-tagged EBNA3C, Flag-tagged IRF8 and Myc-tagged IRF4. Additionally, cells were transfected with GFP expression vector. Transfected cells were selected for 2 weeks with G418 antibiotic. A) 0.1×10^6^ cells from each set of selected samples were plated and cultured for 6 days. Viable cells were counted at indicated time points by trypan blue dye exclusion technique. (B) G418 selected stable cells were harvested, lysed in RIPA buffer and subjected to immunoblot analyses with indicated antibodies. C–D) HEK-293 cells transfected with different combinations of expression plasmids as indicated in previous experiment and selected similarly as described above with G418 antibiotic. After 2 weeks of selection, GFP fluorescence of each plate was scanned by a PhosphorImager and the area of the colonies was calculated by Image J software. The data represented in bar diagram as the average of three independent experiments. E–J) Lentivirus mediated knock down of IRF4 in EBV negative Ramos cells. Knocked down cells were selected with puromycin to make stable cell lines expressing specific si-RNA against IRF4 and control vector. F) The selected stable cells with GFP fluorescence were monitored by fluorescent microscope. G) 50 million Ramos, stable Sh-Ctrl Ramos, Sh-IRF4 Ramos cells were lysed in RIPA buffer and Western blot was performed to check the expression levels of IRF4. E) Stable Sh-Ctrl Ramos, Sh-IRF4 Ramos, cells were transfected with control vector, Flag-IRF8, Myc-EBNA3C and grown in RPMI medium for 36 hrs. Transfected cells were stained with Propidium iodide and subjected to FACS analysis. H) The bar diagram represents the percentage of apoptosis in each transfected set. The results shown are representative of three independent experiments. I–J) 1×10^6^ stable transfected cells were plated and allowed to grow in RPMI media for 6 days. Viable cells were counted by trypan blue dye exclusion method at indicated time points.

To determine the crucial role of EBNA3C and IRF4 on the growth inhibitory property of IRF8, we transfected Ctrl-vector, Myc-EBNA3C, Flag-IRF8, Flag-IRF4 and GFP expression plasmids into HEK-293 cells. Transfected cells were selected by G418 for 2 weeks and cell proliferation rate was determined at different time points. The results demonstrated that the growth inhibitory property of IRF8 was reduced by IRF4 with an additive effect when EBNA3C was included (Fig. 10A). Western blot analysis was performed with those stable transfected cells to monitor the expression levels of both proteins. The results indicated that IRF8 protein expression levels were reduced in the presence of EBNA3C and IRF4 (Fig. 10B). GAPDH Western blot was shown as internal loading control and GFP protein blot were used to demonstrate equal amount of protein lysates from stable transfected cells. We further performed colony formation assays with the same transfected stable cells above. Interestingly, we saw a significant increase in colony numbers when EBNA3C and IRF4 proteins were co-expressed with IRF8 protein in comparison to the set transfected with only IRF8 (Fig. 10C and 10D). These results support our previous results above suggesting that EBNA3C can suppress the growth inhibitory effect of IRF8 in association with IRF4. To further explore the significance of IRF4 expression in EBNA3C-mediated inhibition of IRF8 function, we generated IRF4 knockdown stable Ramos cell lines, which is EBV negative. Lentivirus-mediated transduction was further confirmed by GFP expression (Fig. 10F), and the expression level of knockdown IRF4 was also verified by Western blot analysis. The results demonstrated that the level of IRF4 was knocked down by sh-RNA in comparison with LCL1 or sh-control vector transduced LCL1 cells (Fig. 10G). We then transfected control-vector, Flag-IRF8, Myc-EBNA3C expression vectors in Sh-Ctrl and Sh-IRF4 Ramos cells and subjected them to cell proliferation analysis. Surprisingly, we found that heterologous expression of IRF8 significantly reduced the proliferation of Sh-IRF4 Ramos cells compared with the Sh-Ctrl Ramos cells and that the rate of proliferation was partially restored by EBNA3C (Fig. 10, compare 10I and 10J). Overall, the data demonstrated a critical role for EBNA3C in enhancing the oncogenic effect of IRF4 for promoting B-cell proliferation. To further corroborate these results we extended our study using FACS analysis. The results clearly indicated that IRF4 knockdown Ramos cells showed a higher level of apoptosis compared to the control knockdown cells. Moreover, we observed a substantial level of apoptosis with increased expression of IRF8 in IRF4 knockdown Ramos cells. Interestingly, co-expression with IRF8 and EBNA3C reduced the level of apoptosis (Fig. 10E and 10H). Therefore, EBNA3C co-operates with IRF4 a major regulator of EBV positive cells through downregulation of IRF8.

## Discussion

EBNA3C, one of the EBV essential latent proteins, regulates transcription of several viral and cellular genes. Previous studies showed that EBNA3C also co-operates with EBNA2 to activate the viral LMP1 promoter via interaction with cellular transcription factors, including Spi-1/Spi-B [52]. Additionally, EBNA3C associates with the metastasis suppressor protein Nm23-H1 to regulate the transcription of cellular genes which are critically involved in cell migration and invasion [63]. Recent studies also demonstrated that EBNA3C can interact and stabilize cellular oncoproteins, including c-Myc [11], and the major cell cycle regulatory protein Cyclin D1 [64] to drive B-cell transformation.

IRF4 is an important member of the IRF family of transcription factors, which plays a key role in activation of lymphocytes and generation of antibody producing plasma cells during immune response [65]. Immunohistochemical analysis showed that IRF4 expression was observed in a variety of B-cell lymphomas including Diffuse large B-cell lymphomas (DLBCLs), marginal zone lymphomas (MZLs) and B-cell chronic lymphocytic leukemias (B-CLL) or small lymphocytic lymphomas (SLL) strongly suggesting its involvement with various myeloid and lymphoid malignancies [66]. Notably, IRF4 alone is not sufficient to drive the oncogenic process in B cells, but requires the help of additional factors for the induction of its oncogenic activity [67]. Moreover, the molecular interplay between viral factors and the cellular microenvironment may further determine the specific contributions of IRF4 for driving lymphomagenesis. Among the IRF family members, IRF4 is closely related to IRF8 by sequence homology. Interestingly both IRF4 and IRF8 weakly bind to ISRE in the presence of other DNA binding proteins [53]. In particular, IRF4 forms a complex with IRF8 to regulate the expressions of different cellular genes [67].

Earlier reports have suggested that cellular IRF4 is a key mediator of EBV-induced B cell transformation [43]. However, a lack of expression of the ICSBP/IRF8 gene may add to the progression of lympho-proliferative diseases, as the loss of expression was linked to increased resistance to apoptosis induced by DNA damage or CD95/FAS [68]. Some studies reported that the functions of IRF4 are dependent on the interaction with other cellular proteins [69]. This motivated us to explore the precise interplay between EBNA3C, IRF4 and IRF8 so as to decipher the underlying molecular mechanism.

Our current study showed a direct association between IRF4, IRF8 and EBNA3C. Here, we explored the molecular association between EBNA3C, IRF4 and IRF8 complexes to identify different domains of EBNA3C which regulates the activity of IRF4 and IRF8. Noteworthy, our present investigation demonstrated that EBNA3C binds with IRF4 and IRF8 via the same residues 130–190 within the N-terminal domain. Additionally, we performed different functional assays with an N-terminal deleted mutant of EBNA3C involving IRF4 and IRF8 which ultimately demonstrated a critical regulatory role for this N-terminal domain in cell proliferation and protein stability. Recent genetic studies also suggested that deletion of this specific domain could not support the proliferation of EBV-transformed cells [70]. Our data also supports and re-confirmed this important finding. Interestingly, we identified Spi-1/B like motif in IRF4 and examined the binding affinity of EBNA3C with IRF4 using wild type and Spi-1/B motif deleted mutant. We observed minimal or no binding with Spi-1/B deleted IRF4 and EBNA3C. Our data strongly suggest that this specific Spi-1/B motif permits IRF4 greater flexibility for interacting with EBNA3C. Furthermore, additional studies may require in-depth understanding of the functional significance of Spi-1/B motif. We established that EBNA3C co-localizes with IRF4 and IRF8 proteins in the nuclear compartments from our immunofluorescence assay. These results provide new information which shows that EBNA3C and IRF4 interaction can enhance the stabilization and nuclear accumulation of IRF4 creating a favorable environment important for transformation of EBV-infected cells. Our study further showed that EBV infection of peripheral blood mononuclear cells as well as EBV positive immortalized B-cells increased the stability of IRF4 but not IRF8. However, the mRNA expression level was unchanged, strongly demonstrating that EBNA3C-mediated regulation of IRF4 and IRF8 expression was based primarily on post-translational modification. It was reported that LMP1 was one of the viral factors involved in increased expression of IRF4 in EBV transformed cells [43]. However, the precise molecular mechanism, by which IRF4 contributes to EBV-mediated lymphomagenesis has not yet been delineated. Our study clearly demonstrated a direct role of EBNA3C in regulation of IRF4 and IRF8 expressions independent of other EBNAs or LMP1. Studies in the past have suggested that IRF8 is a target for Ubiquitin-dependent degradation but provided no evidence [71]. Interestingly, ubiquitination assays confirmed that poly-ubiquitination of IRF4 was inhibited by EBNA3C. These findings also correlated with higher expression levels and accumulation of IRF4 in EBV transformed and EBNA3C expressing cells. Interestingly, EBNA3C enhanced the degradation of IRF8 through the Ub-proteasome pathway. Our study now provides validation for a major role of EBNA3C in contributing to IRF4-mediated IRF8 down regulation in EBV transformed B-lymphocytes. Our future study will address the underlying mechanism of IRF4 mediated enhanced poly-ubiquitination of IRF8, involving EBNA3C which will provide clues to further our understanding of the contribution of transcription factors in EBV-mediated oncogenesis. Our knockdown assays confirmed that inhibition of IRF8 was critically linked to EBNA3C-mediated up-regulation of IRF4 thus contributing to its oncogenic activity and overall EBV-mediated B-cell immortalization. This molecular mechanism is important and opens new therapeutic avenues by which IRF4 can be targeted as a potential candidate for neutralizing EBV-mediated B-cell malignancies.

Recent reports suggested that siRNA mediated IRF4 knockdown inhibits Hodgkin Lymphoma cell proliferation and survival [72]. Additionally, IRF4 also possesses anti-apoptotic activities and the regulation of IRF8 by IRF4 may contribute to the biological functions of IRF4 in the context of EBV transformation. In our current study, IRF4 knockdown sufficiently inhibited the proliferation of EBV transformed cells and also augmented the level of etoposide induced apoptosis. Moreover, inhibition of cellular proliferation was directly associated with increased IRF8 expression in IRF4 knockdown LCL1. Importantly, some downstream target molecules of IRF4 such as, c-Myc, Cyclin B1, Cyclin-dependent kinase 6 which are directly associated with cell proliferation, apoptosis and cell-cycle regulation, were found downregulated upon IRF4 knockdown. Interestingly, re-introduction of IRF4 in stable IRF4 knockdown LCL1 cells restored the expression of those downstream signaling molecules. These findings thus infer that EBNA3C-mediated upregulation of IRF4 leads to activation of its downstream signaling cascades which facilitates a favorable condition for B-cell transformation. Few earlier reports provided definitive evidence for the role of IRF8 in inducing apoptosis [73]. Specifically, in humans, IRF8 expression was high in normal hematopoietic cells but impaired in myeloid leukemia [41]. Moreover, IRF8 expression was extremely low or undetectable in 79% of chronic myelogeneous leukemia (CML) patients and 66% of acute myeloid leukemia (AML) patients [41]. Mouse model systems, with a null mutation in IRF8 a myeloproliferative syndrome was observed with marked expansion of undifferentiated myeloid cells that can progress to significant and fatal blast crisis of human CML [39]. On the basis of these previous reports we hypothesized that IRF8 may function as a tumor suppressor in EBV associated B-cell lymphomas. Our studies using EBV negative stable IRF4 knockdown Ramos cells showed a significant level of apoptosis, and a reduction of cell proliferation upon IRF8 expression. Expectedly, cell proliferation was restored and the apoptotic level reduced with transfection of EBNA3C. These findings also correlated with our colony formation assays and cell proliferation assays where we showed that EBNA3C suppressed the growth inhibitory effect of IRF8 in association with IRF4. Furthermore, knockdown of oncogenic IRF4 induced the tumor suppressive activities of IRF8. This cemented our findings that EBNA3C and IRF4 do play a critical role in EBV-infected B-cell proliferation by downmodulating IRF8.

**Figure 11:**
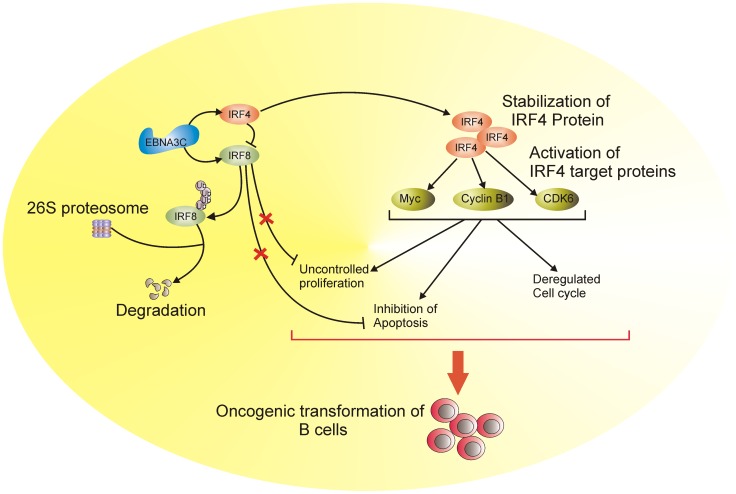
A schematic which illustrates the contribution of EBNA3C to oncogenic transformation of B-cells through stabilization of IRF4 and degradation of IRF8 resulting in activation of IRF4 targeted proteins. EBNA3C interacts with IRF4 and IRF8 proteins and enhances the stability of IRF4 through inhibition of the poly-ubiquitination process. However, in the presence of EBNA3C, IRF4 targets IRF8 and facilitate the subsequent degradation of IRF8. EBNA3C can also contribute to B-cell proliferation by activating IRF4 downstream signaling as well as inhibiting the growth suppressive and apoptosis inducing properties of IRF8. This molecular strategy potentiates oncogenic activity of IRF4 in EBV transformed B-cell proliferation.

In our study, we now focus on the intermolecular interactions and regulatory roles of EBNA3C associated with IRFs. This study provides new evidence which shows a complex of EBNA3C with IRF4 and IRF8 in EBV transformed cells. We have identified the specific interaction domain of EBNA3C for IRF4 and IRF8 binding, and also showed that the specific interaction of EBNA3C and IRF4 ultimately led to enhanced stabilization of IRF4. Additionally, this new role for EBNA3C in stabilizing IRF4 and at the same time enhancing ubiquitin-mediated IRF8 degradation is a novel finding which adds to the overall depth of our understanding of the range of molecular activities which contributes to EBV mediated lymphomagenesis (Fig. 11). Furthermore, siRNA mediated knockdown of IRF4 results in apoptosis in EBV-transformed cells and inhibited proteasome-mediated degradation of IRF8. These knockdown experiments of EBNA3C and IRF4 further strengthened their critical roles in EBV-mediated B-cell proliferation by suppressing the apoptotic process. The downstream molecular targets of IRF4, which include, c-Myc, Cyclin B1, Cyclin-dependent kinase 6 are directly linked to different cellular functions and the deregulated expression of these molecules are implicated in EBV-mediated pathogenesis. Our findings thus provide another insight into the role of EBNA3C expressed in EBV-infected B-cells and its association with critical IRF family members which contributes to viral induced cell transformation. This study may provide novel therapeutic targets to treat and prevent EBV- associated lymphomas.

## Materials and Methods

### Plasmids, antibodies and cell lines

Plasmids expressing full length EBNA3C or its truncations such as 1–365, 366–620, 621–992, and different N-terminal truncated or deleted mutants with C-terminal Flag or Myc-tagged, catalytic domain mutant Myc-tagged EBNA3C, GST-EBNA3C with single or double point mutations and EBNA3C tagged with GFP have been mentioned previously [11], [55], [56], [74]. Flag-tagged human IRF4 construct was generated by using Invitrogen cDNA clone pOTB7-IRF4. PCR amplified insert was digested with EcoRI/NotI and ligated into pA3F and pA3M vectors. Flag or Myc-tagged constructs expressing different truncations of IRF4 and IRF8 domains, Spi-1/B motif deleted Flag-tagged IRF4 were generated by PCR mutagenesis. Retroviral pMSCV-IRF8-IRES-EGFP plasmid was kindly provided by Ben-Zion Levi (Technion-Israel Institute of Technology). This construct was used to generate human pA3F-IRF8 by cloning the PCR amplified insert into Myc and Flag-tagged vectors. pGEX-IRF4 and IRF8 constructs were cloned by using pA3F-IRF4 and IRF8 constructs as templates for the PCR amplification. PCR amplified gene products were inserted in pGEX2TK vectors (GE Healthcare Biosciences, Pittsburgh, PA). pCDNA3-HA-Ub construct was kindly provided by George Mosialos (Alexander Fleming Biomedical Science research center, Vari, Greece) pGIPZ (Open Biosystems, Inc. Huntsvillle, AL) was used as the sh-RNA vector. Plasmids used for lentiviral packaging were described previously [75].

Antibodies of IRF4 (H-140), IRF8 (S-15), Ub (FL-76), PARP-1 (F-2) were purchased from Santa Cruz Biotechnology, Inc (Santa Cruz, CA) GAPDH antibody was purchased from US-Biological Corp. (Swampscott, MA). Flag (M2)-epitope, anti-mouse antibody was purchased from Sigma-Aldrich Corp. (ST. LOUIS, MO). Other antibodies to mouse anti-Myc (9E10), anti-Hemaggutinin (12CA5), A10 were previously described [75].

HEK-293 (human embryonic kidney cell line) was kindly provided by Jon Aster (Brigham and Woman's Hospital, Boston, MA, USA). Cells were grown in Dulbeccoo's modified Eagle's medium (DMEM; Hyclone, Logan, UT) supplemented with 5% fetal bovine serum (FBS; Hyclone, Logan, UT), 25 U/ml penicillin 50 µg/mal streptomycin, 2 mM L-glutamine (Hyclone, Logan, UT). MutuI and MutuIII cells were provided by Yan Yuan (School of Dental Medicine, university of Pennsylvania, Philadelphia, PA). EBV negative BL cells BJAB, Ramos, DG75, BL41 and B95.8 infected BL41 were kindly provided by Elliot Kieff (Harvard medical School, Boston, MA). BJAB stably expressing EBNA3C (BJAB7, BJAB10) were previously described [76]. In vitro-transformed EBV positive lymphoblastoid cell lines (LCL1, LCL2), EBV positive P3HR1, Jijoye cells and other B-cells were maintained in RPMI 1640 media (Hyclone, Logan, UT). The above mentioned cell lines were incubated at 37°C in humidified 5% Co_2_ environment.

### Transfections

Adherent HEK-293 cells and B-cells were transfected by electroporation system with Bio-Rad Gene Pulser II electroporator using 0.4 cm gap cuvette and cells were electroporated at 210 V and 975 µF (for HEK-293) or 220 V and 975 µF (for DG75). After transfection, cells were grown in 10 ml of complete media in 100 mm Petri dish.

### Infection of Peripheral Blood Mononuclear cells (PBMC) with BAC GFP-EBV

PBMC were obtained from University of Pennsylvania Immunology core donated by the healthy donors. As mentioned earlier [48], the Core maintains an IRB approved protocol in which declaration of Helsinki protocols were followed and each donor gave written, informed consent. 10 million PBMC were mixed well with BAC-GFP EBV supernatant in 1 ml of RPMI 1640 media supplemented with 10% FBS. Cells were incubated for 4 hrs at 37°C. Cells were centrifuged and cell pellet was re-suspended with 2 ml of complete RPMI 1640 media. EBV GFP expression was observed by using fluorescence microscope. The cells were harvested after 0, 2, 4, 7, 15 days of post-infection.

### Immunoprecipitation and Western blot analysis

Cells were harvested and washed with 1X Phosphate Buffered Saline (PBS). 0.5 ml radioimmunoprecipitation assay (RIPA) buffer (0.5% NP-40, 10 mM Tris pH 7.5, 2 mM EDTA, 150 mM NaCl supplemented with 1 mM phenylmethylsulphonyl fluoride (PMSF), Aprotinin, pepstatin and leupeptin were used as 1 µg/ml for cell lysis. Lysates were then pre-cleared with normal mouse or rabbit serum by rotating with 30 µl of Protein-A and Protein-G (1∶1 mixture) -conjugated Sepharose beads for 1 hr at 4°C. Approximately 5% of the protein lysate was saved as input. 1 µg of specific antibody was used to capture the specific protein of interest by overnight rotation at 4°C. Immuno–complexes were precipitated with 30 µl of Protein-A and Protein-G-conjugated Sepharose beads. The immune-precipitated samples were washed with RIPA buffer.

Protein samples were boiled in laemmli [77] buffer and resolved by SDS-PAGE and transferred to nitrocellulose membrane (0.45 µm). The membranes were probed with appropriate antibodies and scanned by Odyssey imager (LiCor Inc., Lincoln, NE) Quantitation and image analysis were performed by Odyssey infrared imaging System application software.

### Purification of GST fusion proteins and GST pull-down assay

Glutathione S-transferase (GST), GST-IRF4, GST-IRF8 constructs were transformed in *Escherichia coli* BL21 cells and single colonies were grown in 3 ml of Luria broth overnight culture supplemented with 100 µg/ml ampicillin. 1 ml of this overnight culture was used to inoculate 500 ml of culture. When OD_600_ of that culture reached approximately 0.6, Isopropyl-β-D-thiogalactopyranoside (IPTG) induction was performed with 0.5 mm concentration for 12 h at 30°C. Bacterial pellet was washed with STE buffer (100 mM NaCl, 10 mM Tris, and 1 mM EDTA, pH 7.5), and resuspended with 3 ml of NETN buffer (0.5% NP-40, 100 mM NaCl, 20 mM Tris, 1 mM EDTA, pH 8.0) supplemented with protease inhibitors and incubated 15 min on ice. 150 µl of 1 M dithiothreitol (DTT) and 1.8 ml of 10% Sarkosyl were added in STE buffer and the suspension was sonicated. The post-sonicated samples were centrifuged at 12,000×g for 10 min at 4°C. The supernatant was transferred into a fresh tube. 3 ml of 10% Triton X-100 in STE buffer and 200 µl of GST beads were added to the samples were rotated at 4°C for overnight. Purified GST protein samples were washed with NETN buffer supplemented with protease inhibitors. Purified protein expression was checked by SDS-PAGE. For GST pull-down assay, BJAB, BJAB7, BJAB10, LCL1, LCL2 cell lysates were incubated with GST fusion proteins and control GST protein. Protein samples were washed with Binding Buffer (1X PBS, 0.1% NP-40, 0.5 mM DTT, 10% glycerol, protease inhibitors) and resolved with 8% SDS-PAGE. Western blot was performed with A10 antibody.

### Immunofluorescence

HEK-293 cells were plated on coverslips and different expression plasmids were by Lipofectamine 2000 transfection reagents (Invitrogen, Carlsbad, CA). After 36 hrs of post-transfection, transfected cells were fixed with 3% paraformaldehyde (PFA) with 0.1% Triton X-100 for 20 mins at room temperature. Fixed cells were washed with 1X PBS and 1% Bovine serum albumin was used for blocking. Flag-tagged IRF4 were detected by anti-flag (M2) antibody and GFP-tagged EBNA3C was detected by GFP-fluorescence. BJAB, BJAB7, BJAB10, LCL1 and LCL2 cells were semi-air-dried on slides and fixed as mentioned above. To check endogenous expressions of EBNA3C, and IRF4, specific antibodies were used. Primary antibodies were diluted in blocking solution. Fixed cells were incubated with these primary antibodies for overnight at 4°C. Cells were washed with 1X PBS and incubated with secondary antibodies (1∶1000) for 1 hr at room temperature. Nuclear staining was performed with DAPI (4′,6′,-diamidino-2-phenylindole; Pierce, Rockford, IL). Cells were washed in 1X PBS and mounted with antifade mounting medium. The slides were observed by Fluoview FV300 confocal microscope. Images were analyzed by FLUOVIEW software (Olympus Inc., Melville, NY).

### RNA isolation and Real-time quantitative PCR

Cells were washed with ice-cold 1X PBS prior to RNA isolation. RNA extraction was performed by Trizol reagent (Invitrogen, Inc., Carlsbad, CA) according to manufacturer's protocol. cDNA was prepared by using Superscript II reverse transcriptase kit (Invitrogen, Inc., Carlsbad, CA) according to the instructions of the manufacturer. The primers for IRF4, IRF8, and EBNA3C were 5′-CAAGAGCAATGACTTTGAGG-3′ and 5′-TGGGACATTGGTACGGGAT-3′ [78], 5′-CAGTGGCTGATCGAGCAGATTGA-3′ and 5′-ATTCACGCAGCCAGCAGTTGCCA-3′ [41], 5′-AGAAGGGGAGCGTGTGTTGT-3′ and 5′-GGCTCGTTTTTGACGTCGGC-3′ respectively. Primers for GAPDH were 5′-TGCACCACCAACTGCTTAG-3′ and 5′-GATGCAGGGATGATGTTC-3′ [64]. Quantitative real-time PCR analysis was performed by using SYBER green Real-time master mix (MJ Research Inc., Waltham, MA). A melting curve analysis was performed to determine the specificity of the products and the values for the relative quantitation were calculated by threshold cycle method. Each sample was examined in triplicate.

### Stability assay

Transiently transfected HEK-293 cells and B-cells (BJAB, BJAB7, BJAB10, LCL1, and LCL2) were treated with protein synthesis inhibitor cyclohexamide (CalBiochem, Gibbstown, NJ) as 40 µg/ml concentration. Cells were harvested in different time points. Cells were lysed with RIPA buffer and protein samples were used for Western blot analysis. Protein band intensities were quantified by Odyssey 3.0 software.

### In vivo poly-ubiquitination assay

HEK-293 cells were transfected with control-vector, Flag-IRF4, Flag-IRF8, HA-Ub, Myc-EBNA3C expression plasmids by electroporation. After 36 hours post-transfection, cells were pre-treated with 20 µM concentration of MG132 (Enzo Life Sciences International, Inc., Plymouth Meeting, PA) for additional 6 hours. Cell lysates were prepared and specific proteins were immunoprecipitated by using specific antibodies. Immunoprecipitated samples were resolved by SDS-PAGE. The status of ubiquitination was determined by HA-specific (12CA5) antibody.

### Lentiviral sh-RNA mediated gene silencing

The sense strand of IRF4 shRNA and EBNA3C shRNA sequences are 5′- tcgagtgctgttgacagtgagcgaGCATGAACCTGGAGGGCGGtagtgaagccacagatgtaCCGCCCTCCAGGTTCATGC gtgcctactgcctcggaa-3′ [43] and 5′- tcgagtgctgttgacagtgagcgaCCATATACCGCAAGGAATAtagtgaagccacagatgtaTATTCCTTGCGGTATATGGgtgcctactgcctcggaa-3′ [64] respectively. Here, upper-case letters designate either IRF4 or EBNA3C target sequences and lower-case letters specify hairpin and sequences which are required for the directional cloning in pGIPZ vector. These single stranded oligonucleotides were individually cloned into the pGIPZ vector using XhoI and MluI restriction sites. Also, a control shRNA sequence; 5′-TCTCGCTTGGGCGAGAGTAAG-3′ (Dharmacon Research, Chicago, IL) was used to make Sh-Ctrl vector which lack the complementary sequences in the human genome.

For production of lentivirus, 2×10^6^ HEK 293T cells were grown in DMEM media with 10% FBS for 24 hrs prior to transfection. Total 20 µg of plasmid expression vector was used for the transfection of each set, including 1.5 µg of pCMV-VSV-G, 3 µg of pRSV-REV, 5 µg of pMDLg/Prre (Addgene, Inc., Cambridge, MA), and 10.5 µg of lentiviral vector plasmid. For precipitation, plasmids were added to a final volume of 438 µl of sterile H_2_0 and 62 µl of 2 M CaCl_2_, and solutions mixed well, then 500 µl of 2×HEPES-buffered saline added. Each transfection set was incubated at room temperature for 30 min. Before transfection, chloroquine was added to the 10 ml of media with a final concentration of 25 µM for 5 min. The media was replaced with DMEM supplemented with 10% FBS and 10 mM HEPES, and 10 mM sodium butyrate after 12 hrs of incubation. Again, the media was replaced after 10 hrs by DMEM supplemented with 10% FBS with 10 mM HEPES. To collect virus, the conditioned media was collected four times at 12 hrs interval. Conditioned medium was filtered through cellulose acetate filters (0.45 µm) and stored in ice. The virus was concentrated by centrifuging the medium at 70,000×g for 2.5 hrs. The concentrated virus was re-suspended in RPMI medium and the virus used to infect 10^6^ LCL1 cells with Polybrene as 20 µM/ml concentration. After 72 hrs of incubation, puromycin antibiotic was added as 2 µg/ml concentration for selection. To check the rate of selection, GFP-immunofluorescence was observed by Olympus 1X71 microscope with 560 nm excitation and 645 nm emission filters. Puromycin selected cells were grown up to 80% confluence and the expression levels of target proteins were checked by western blot analysis.

### Proliferation assay

HEK-293 cells were transfected with different expression vectors by electroporation. Transfected cells were grown in DMEM. Cells were selected with 1000 µg/ml G418 antibiotic (Invitrogen, Inc., Carlsbad, CA) for 2-weeks. After selection, cell lysates were prepared by RIPA buffer and protein expression was checked by Western blot analysis. From each transfected set, 0.1×10^6^ cells were plated and allowed them to grow for 6 days. In case of stable knockdown LCL1 cells, the same number of cells were plated and grown in RPMI media. Viable cells were counted in specific time points with Trypan Blue dye exclusion technique. All experiments were performed in triplicates.

### Colony formation assay

5×10^6^ Human kidney embryonic cells were transfected with Ctrl-vector, Flag-IRF8, Flag-IRF4, Myc-EBNA3C, GFP-control vector by electroporation and allowed to grow in DMEM with G418 as 1 mg/ml concentration. After 2-weeks of selection, GFP fluorescence of each plate was scanned by PhosphorImager (Molecular Dynamics, Piscataway, NJ) and the area of the colonies measured by using Image J software (Adobe Inc., San Jose, CA). Three independent experiments were performed to take average data.

### Apoptosis assay

Control vector, IRF4 knockdown LCL1 cells were treated with or without etoposide (MP Biomedicals, LLC) overnight and cells were harvested and washed with 1xPBS three times. Flag-IRF8 co-transfected with Ctrl-vector and Myc-EBNA3C in IRF4 knockdown and sh-control vector transfected stable Ramos cell lines. These cells were washed with 1X PBS and fixed with 70% cold ethanol. Cells were kept at 4°C until used for FACS analysis. Fixed cells were stained with PBS containing 10 µg/ml of propidium iodide (PI), 250 µg/ml of RNase A (Sigma) and 0.05% of Triton X-100 for 1 hr at room temperature in dark. Stained cells were analyzed on FACScalibur cytometer and Cellquest software (Becton-Dickinson Inc., San Jose, CA).

### Statistical analysis

Data here are represented as mean values with standard errors of means (SEM). 2-tailed student's t-test was performed to evaluate the significance of differences in the mean values. A P-value of <0.05 was considered as statistically significant.

### Accession numbers

Homo sapiens interferon regulatory factor 4 (IRF4)-GenBank: BC015752.1, Homo sapiens interferon regulatory factor 8 (IRF8)-NCBI Reference Sequence: NM_002163.2, Epstein-Barr virus (EBV) genome, strain B95-8- GenBank: V01555.2, IRF4 protein (Homo sapiens) - GenBank: AAC50779.1, IRF8 protein (Homo sapiens)-NCBI Reference Sequence: NP_002154.1, EBNA3C protein- UniProtKB/Swiss-Prot: P03204.1, Spi-1 protein- UniProtKB/Swiss-Prot: SPI1_HUMAN, P17947, Spi-B protein- UniProtKB/Swiss-Prot: SPIB_HUMAN, Q01892.

## Supporting Information

Figure S1 Quantitation of Irf4 and Irf8 mRNA expression in EBV positive and EBNA3C expressing cells. Total RNA was isolated from BAC-GFP EBV infected PBMC cells (at different time intervals), DG75, BJAB, LCL1, LCL2, stable EBNA3C expressing BJAB cells (BJAB7, BJAB10) and subjected to quantitative real-time PCR analysis to detect EBNA3C, IRF4, IRF8 mRNA levels. For IRF4 and IRF8 transcripts, P-values of the mean differences for A) 2, 4, 7, 15 days of BAC-GFP EBV infected PBMC, compared with 0 day are 0.8075, 0.7157, 0.6666, 0.6913 and 0.3355, 0.4777, 0.4226, 0.7418 respectively. B) The P-values of the mean differences for LCL1, LCL2 compared with BJAB for IRF4 transcripts are 0.2495, 0.0954 respectively and that for IRF8 transcripts are 0.0719, 0.4226 respectively. C) Similarly, the P-values of the mean differences for BJAB7, BJAB10 cells are 0.1844, 0.1917 for IRF4 and 0.4226, 0.8075 for IRF8 compared with BJAB. D) Real-time PCR analysis was performed to check EBNA3C transcript level in EBV transformed LCL1, LCL2 compared with EBV-negative DG75 and BJAB. The P-values of the mean differences for LCL1, LCL2 are 0.0168, 0.0169 compared with DG75 and 0.0165, 0.0167 compared with BJAB respectively. The experiment was performed in triplicate sets and the data is represented here as the difference in the quantity of specific transcripts to the quantity of control GAPDH transcript. The error bars indicate standard deviations from three independent experiments. Here, p-value of <0.05 was considered as statistically significant.
(TIF)

Figure S2 LMP-1 independent induction of IRF4 protein expression in EBV-positive Burkitt 's lymphoma cell lines. 50 million P3HR1, Jijoye cells were subjected to Western blot analysis using A10, S12, IRF4, GAPDH antibodies. The IRF4 protein expression level was found similar in these two cell lines.
(TIF)

Figure S3 EBNA3C binds with IRF4 and IRF8 through its N-terminal domain. A) The schematic diagram represents various structural and interactive domains of EBAN3C and summarizes the binding affinities between different domains of EBNA3C with IRF4 and 8. +, binding; −, no binding. B) The schematic shows the alignment of EBNA3A, EBNA3B and EBNA3C 130–159 amino acids. Functionally conserved residues were indicated by asterisks. Specific single or double point mutations were introduced in this region indicated by boxes.
(TIF)

Figure S4 IRF4 knockdown in EBV transformed LCL1 cells. A) Lentivirus mediated delivery of short hairpin RNA (sh-RNA) vectors knock down IRF4 in EBV transformed LCL1 cells. Knocked down cells were selected with puromycin to make stable cell line expressing specific si-RNA against IRF4 along with control vector. The GFP fluorescence of selected cells was observed by fluorescence microscope. B) 50 million different clones of stable Sh-IRF4, Sh-Ctrl, LCL1 cells were harvested and cell lysates were prepared by RIPA buffer. Western blot analysis was performed to show the expression levels of A10, IRF4 and GAPDH.
(TIF)

Figure S5 EBV transformed and EBNA3C expressing B cells are resistant to etoposide induced cell killing. 1×10^6^ EBV negative BJAB, DG75, EBV transformed LCL1, LCL2, EBNA3C expressing BJAB7, BJAB10, Sh-Ctrl, Sh-EBNA3C transfected stable LCL1 cells were treated with or without etoposide (10 µM) and allowed to grow in RPMI media. Viable cells were counted in different time points by Trypan Blue dye exclusion technique. All experiments were performed three times in triplicates. Here, we observed that EBV negative cells were more sensitized to etoposide induced cell death. On the other hand, EBV transformed and more specifically EBNA3C expressing cells showed enhanced proliferation. Moreover, the cellular proliferation rate was not altered over the indicated time periods upon etoposide treatment. In case of etoposide treated stable EBNA3C knockdown cells, cell proliferation was significantly reduced.
(TIF)

Figure S6 IRF4 knockdown enhances apoptosis in EBV transformed cells treated with etoposide. EBV transformed LCL1 cells were subjected to lentivirus mediated stable transduction by introducing short hairpin RNA (sh-RNA) to knockdown Irf4. Sh-Ctrl RNA also transduced for control set. Stable knockdown cells were treated with etoposide drug for different time points. Next, cells were harvested and pelleted by centrifugation at 1000 RPM (129 g) for 5 minutes. Cell pellets were washed with 1 ml of cold PBS and cell pellets were resuspended in 25 µl of cold PBS and 2 µl of EB/AO (ethidium bromide/acridine orange) dye mix. 10 µl of stained suspension were placed on clean slide and covered with coverslip. Cells were observed and counted by using fluorescence microscope [79]. Experiments were done in triplicates by counting a minimum of 100 total cells each. The data shown here indicates that etoposide treatment significantly enhanced the apoptosis in IRF4 knockdown stable EBV transformed LCL1 cells, compared with the control vector transfected cells.
(TIF)

Figure S7 EBNA3C and IRF4 silencing promotes apoptotic induction in EBV transformed Lymphoblastoid cells. Apoptosis is potentially involved in regulation of cellular proliferation under a wide range of virus-induced pathogenic activities. Importantly, DNA fragmentation is one of the hallmarks of apoptosis [80]. In order to determine if apoptotic events contributed to the reduction in growth rate upon IRF4 knockdown cells, DNA fragmentation assay was performed. 4×10^6^ Sh-Ctrl, Sh-EBNA3C, Sh-IRF4 stable LCL1 cells were collected in 1.5 ml eppendorf tube after washing with 1X PBS Next, cell pellet re-suspend with 0.5 ml 1X PBS and 55 µl of Triton X-100 lysis buffer (40 ml of 0.5 M EDTA, 5 ml of 1 M trisCl buffer pH 8.0, 5 ml of 100% Triton X-100, 50 ml of H_2_O) was added for 20 min on ice. Tubes were centrifuged at 4°C at 12,000 g for 30 min. Samples were transferred to new tubes and supernatants was extracted by using 1∶1 mixture of phenol: chloroform. DNA precipitation was performed by adding in two equivalence of cold ethanol and one tenth equivalence of Sodium Acetate. DNA pellet was re-suspended with 30 µl of de-ionized water-RNase solution (0.4 ml of water with 5 µl of RNase solution) and 5 µl of loading buffer. Samples were incubated for 30 min for 37°C. DNA samples were run by 1.2% gel at 5 V for 5 min before increasing to 100 V. Interestingly, we observed a substantial amount of DNA fragmentation upon sh-RNA based knockdown of IRF4 and EBNA3C.
(TIF)
