# Expression of Concern: The EBV latent antigen 3C inhibits apoptosis through targeted regulation of interferon regulatory factors 4 and 8

**DOI:** 10.1371/journal.ppat.1013777

**Published:** 2025-12-16

**Authors:** The PLOS Pathogens Editors

Following the publication of this article [1], concerns were raised regarding results presented in Figs 3, 9, and 10. Specifically;

- In Fig 3C, the right E3C 1-992 panel appears similar to the left E3C 1-365 panel when flipped.
- The Fig 9E Sh-IRF4 -Etoposide panel appears similar to the Fig 10E Sh-IRF4 Ramos Ctrl-vector panel.
- The Fig 9E Sh-IRF4 + Etoposide panel appears similar to the Fig 10E Sh-IRF4 Ramos Flag-IRF8 panel.

The corresponding author stated that the original underlying data are no longer available. They provided repeat data for the results presented in Fig 3 (S1 File) from later experiments. The corresponding author stated that due to changes in regulations, the repeat experiments were carried out using biotinylated amino acid instead of radioactive S35. The methodology for the repeat experiments is provided in S2 File. The corresponding author also offered to repeat the experiments presented in Figs 9E and 10E, but PLOS does not consider that repeat experiment data alone are sufficient to fully resolve the concerns with Figs 3, 9, and 10 in the absence of the original data underlying the published results.

The *PLOS Pathogens* Editors issue this Expression of Concern to notify readers of the above concerns and to inform readers that these results should be interpreted with caution.

## Supporting information

S1 File Repeat data for the results presented in Fig 3 from later experiments. (JPG)

S2 File Methodology for the repeat data presented in S1 File. (DOCX)
