# Supplementary figures and images for: The EBV Latent Antigen 3C Inhibits Apoptosis through Targeted Regulation of Interferon Regulatory Factors 4 and 8
Source: PLoS Pathog. 2013 May 2;9(5):e1003314. doi: 10.1371/journal.ppat.1003314 (PMC3642079; doi:10.1371/journal.ppat.1003314)

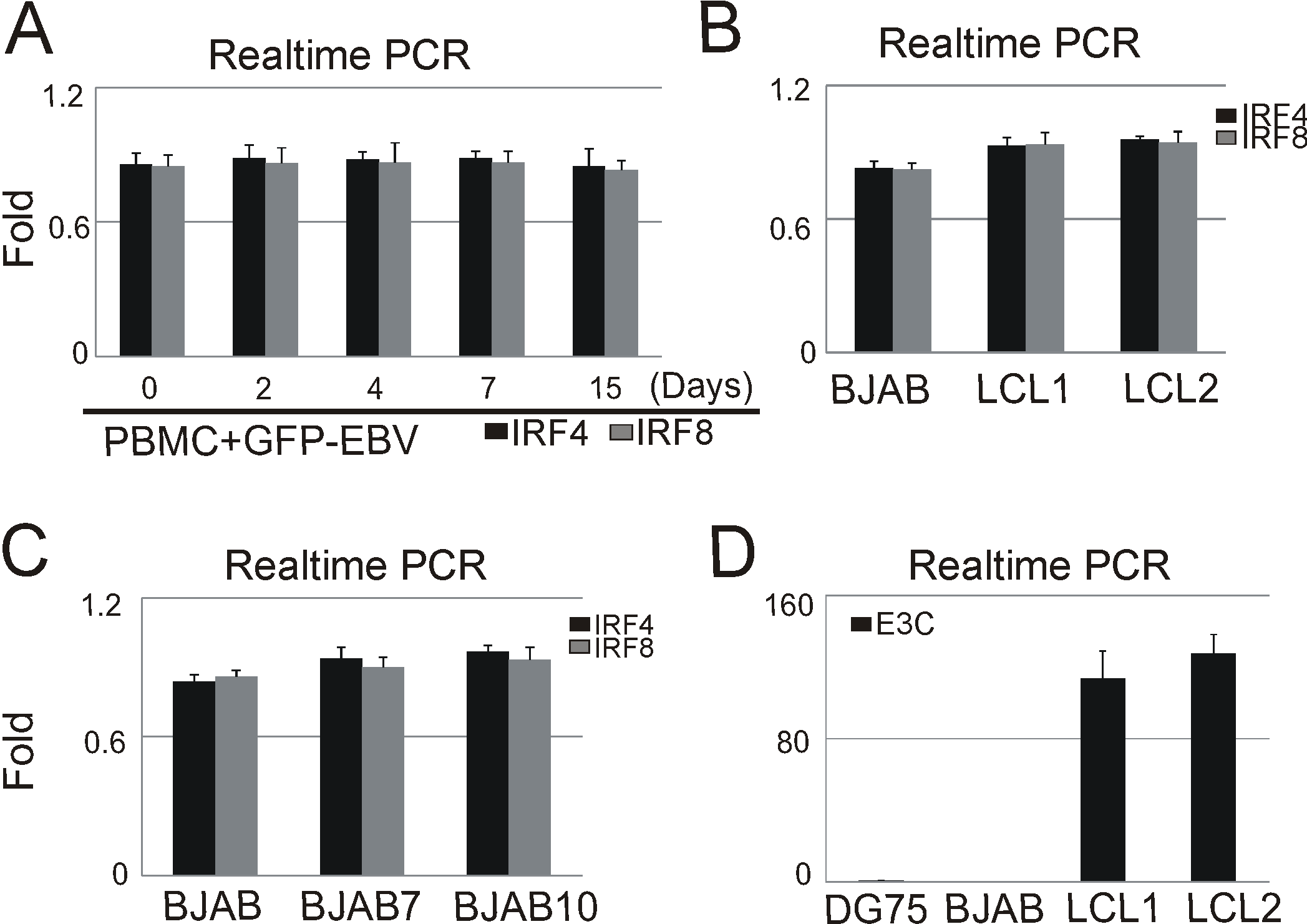

Supplement: Figure S1 — Quantitation of Irf4 and Irf8 mRNA expression in EBV positive and EBNA3C expressing cells. Total RNA was isolated from BAC-GFP EBV infected PBMC cells (at different time intervals), DG75, BJAB, LCL1, LCL2, stable EBNA3C expressing BJAB cells (BJAB7, BJAB10) and subjected to quantitative real-time PCR analysis to detect EBNA3C, IRF4, IRF8 mRNA levels. For IRF4 and IRF8 transcripts, P-values of the mean differences for A) 2, 4, 7, 15 days of BAC-GFP EBV infected PBMC, compared with 0 day are 0.8075, 0.7157, 0.6666, 0.6913 and 0.3355, 0.4777, 0.4226, 0.7418 respectively. B) The P-values of the mean differences for LCL1, LCL2 compared with BJAB for IRF4 transcripts are 0.2495, 0.0954 respectively and that for IRF8 transcripts are 0.0719, 0.4226 respectively. C) Similarly, the P-values of the mean differences for BJAB7, BJAB10 cells are 0.1844, 0.1917 for IRF4 and 0.4226, 0.8075 for IRF8 compared with BJAB. D) Real-time PCR analysis was performed to check EBNA3C transcript level in EBV transformed LCL1, LCL2 compared with EBV-negative DG75 and BJAB. The P-values of the mean differences for LCL1, LCL2 are 0.0168, 0.0169 compared with DG75 and 0.0165, 0.0167 compared with BJAB respectively. The experiment was performed in triplicate sets and the data is represented here as the difference in the quantity of specific transcripts to the quantity of control GAPDH transcript. The error bars indicate standard deviations from three independent experiments. Here, p-value of <0.05 was considered as statistically significant. (TIF) [file ppat.1003314.s001.tif]

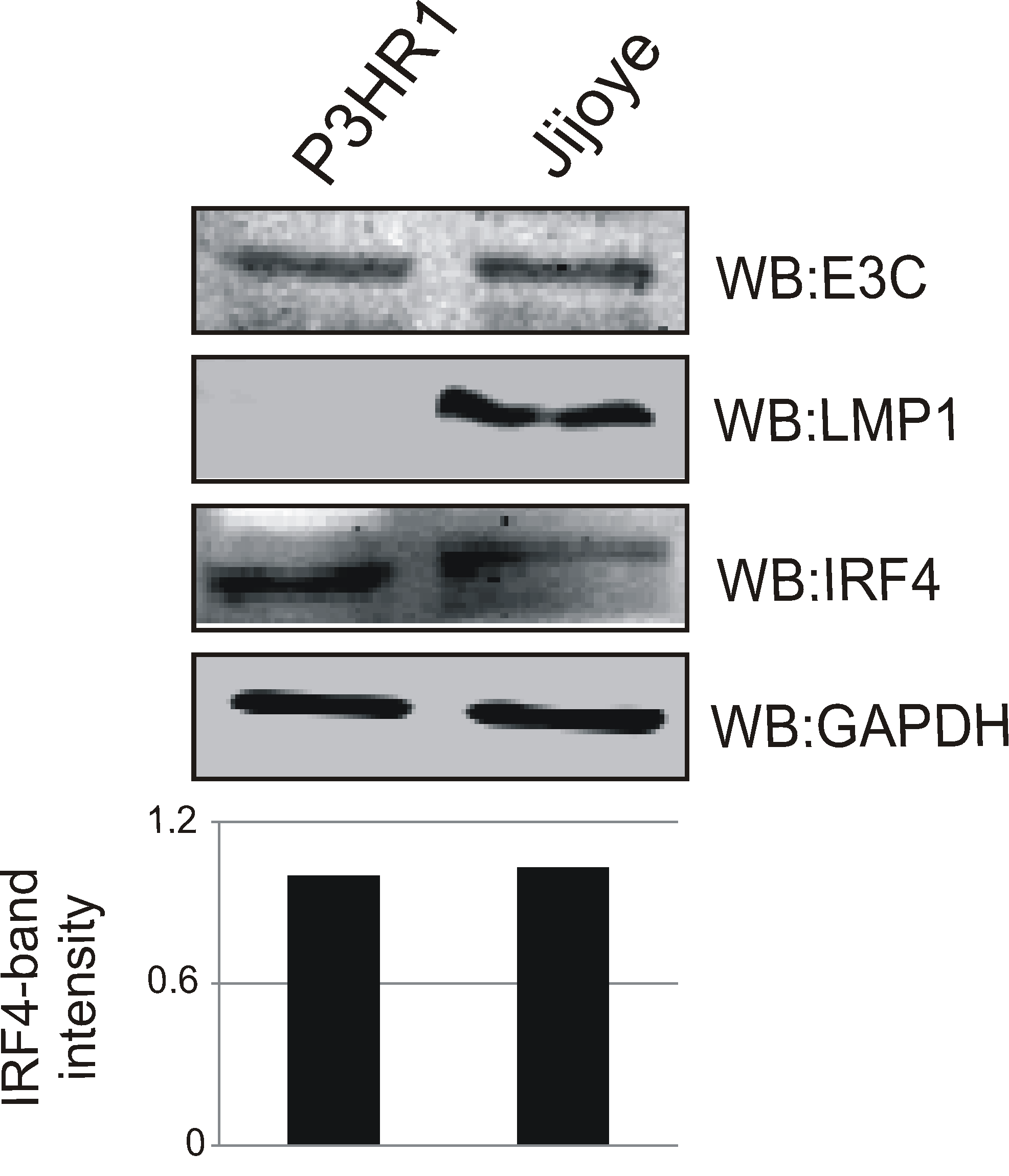

Supplement: Figure S2 — LMP-1 independent induction of IRF4 protein expression in EBV-positive Burkitt 's lymphoma cell lines. 50 million P3HR1, Jijoye cells were subjected to Western blot analysis using A10, S12, IRF4, GAPDH antibodies. The IRF4 protein expression level was found similar in these two cell lines. (TIF) [file ppat.1003314.s002.tif]

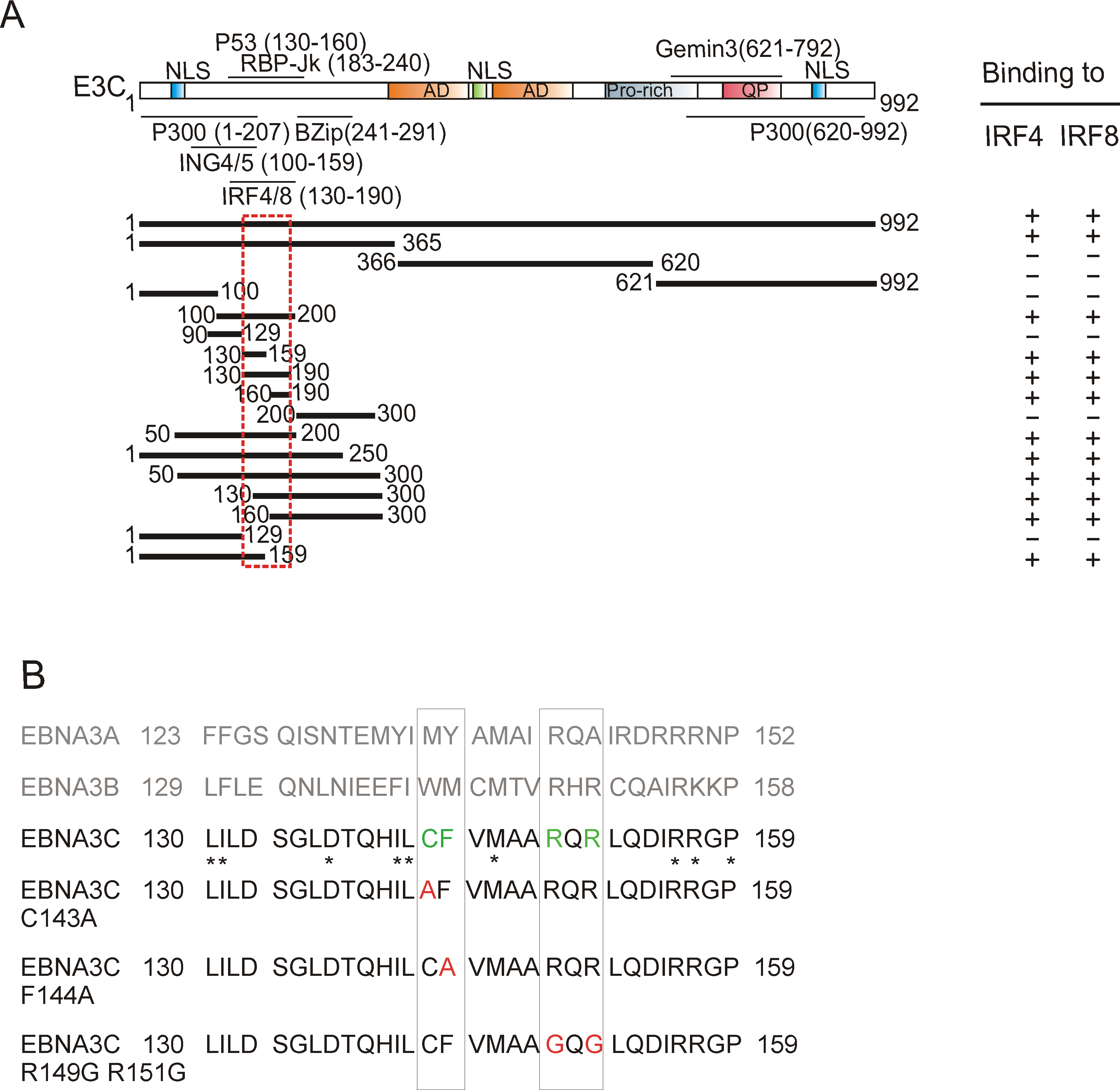

Supplement: Figure S3 — EBNA3C binds with IRF4 and IRF8 through its N-terminal domain. A) The schematic diagram represents various structural and interactive domains of EBAN3C and summarizes the binding affinities between different domains of EBNA3C with IRF4 and 8. +, binding; −, no binding. B) The schematic shows the alignment of EBNA3A, EBNA3B and EBNA3C 130–159 amino acids. Functionally conserved residues were indicated by asterisks. Specific single or double point mutations were introduced in this region indicated by boxes. (TIF) [file ppat.1003314.s003.tif]

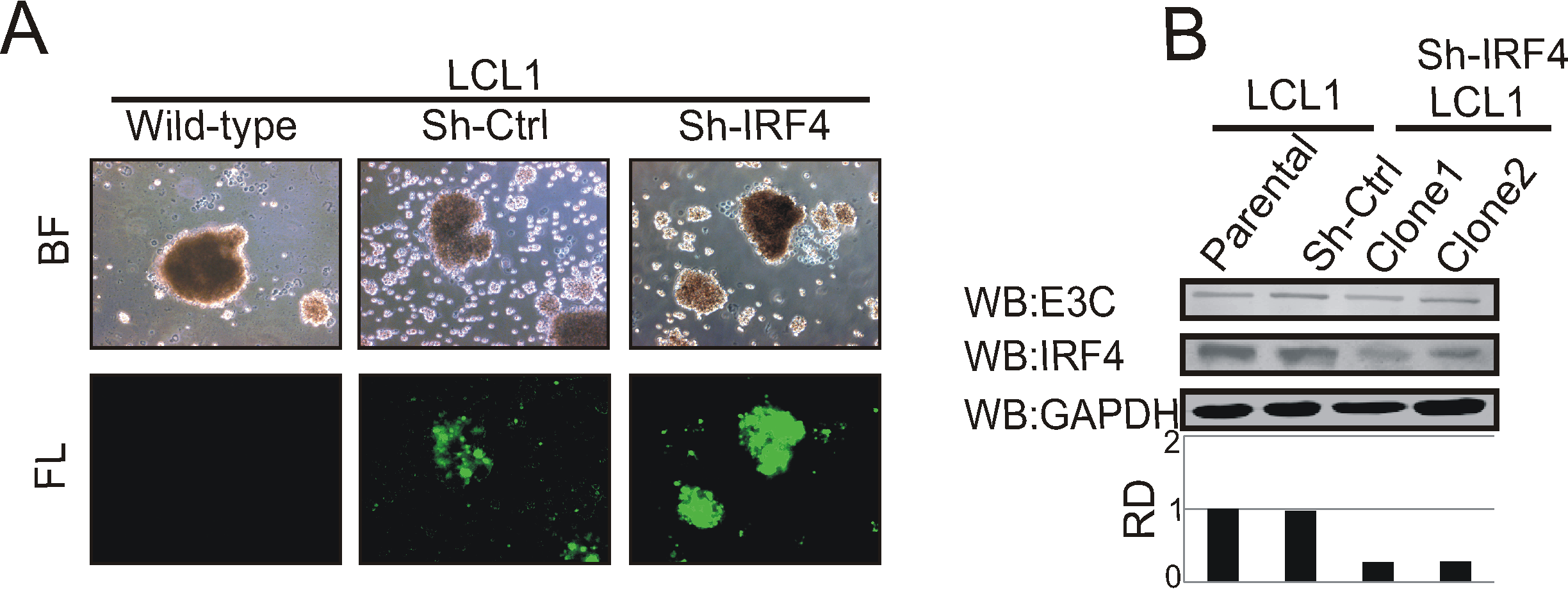

Supplement: Figure S4 — IRF4 knockdown in EBV transformed LCL1 cells. A) Lentivirus mediated delivery of short hairpin RNA (sh-RNA) vectors knock down IRF4 in EBV transformed LCL1 cells. Knocked down cells were selected with puromycin to make stable cell line expressing specific si-RNA against IRF4 along with control vector. The GFP fluorescence of selected cells was observed by fluorescence microscope. B) 50 million different clones of stable Sh-IRF4, Sh-Ctrl, LCL1 cells were harvested and cell lysates were prepared by RIPA buffer. Western blot analysis was performed to show the expression levels of A10, IRF4 and GAPDH. (TIF) [file ppat.1003314.s004.tif]

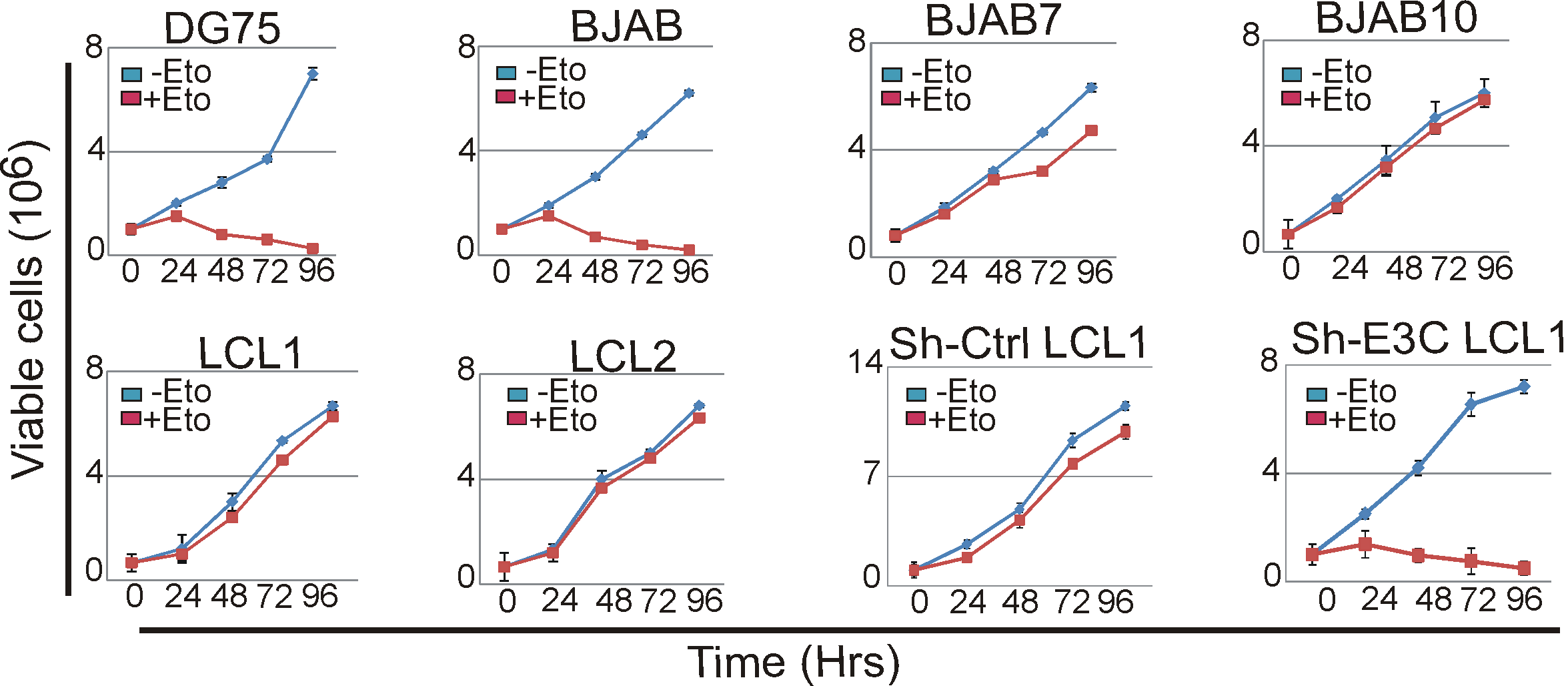

Supplement: Figure S5 — EBV transformed and EBNA3C expressing B cells are resistant to etoposide induced cell killing. 1×10^6^ EBV negative BJAB, DG75, EBV transformed LCL1, LCL2, EBNA3C expressing BJAB7, BJAB10, Sh-Ctrl, Sh-EBNA3C transfected stable LCL1 cells were treated with or without etoposide (10 µM) and allowed to grow in RPMI media. Viable cells were counted in different time points by Trypan Blue dye exclusion technique. All experiments were performed three times in triplicates. Here, we observed that EBV negative cells were more sensitized to etoposide induced cell death. On the other hand, EBV transformed and more specifically EBNA3C expressing cells showed enhanced proliferation. Moreover, the cellular proliferation rate was not altered over the indicated time periods upon etoposide treatment. In case of etoposide treated stable EBNA3C knockdown cells, cell proliferation was significantly reduced. (TIF) [file ppat.1003314.s005.tif]

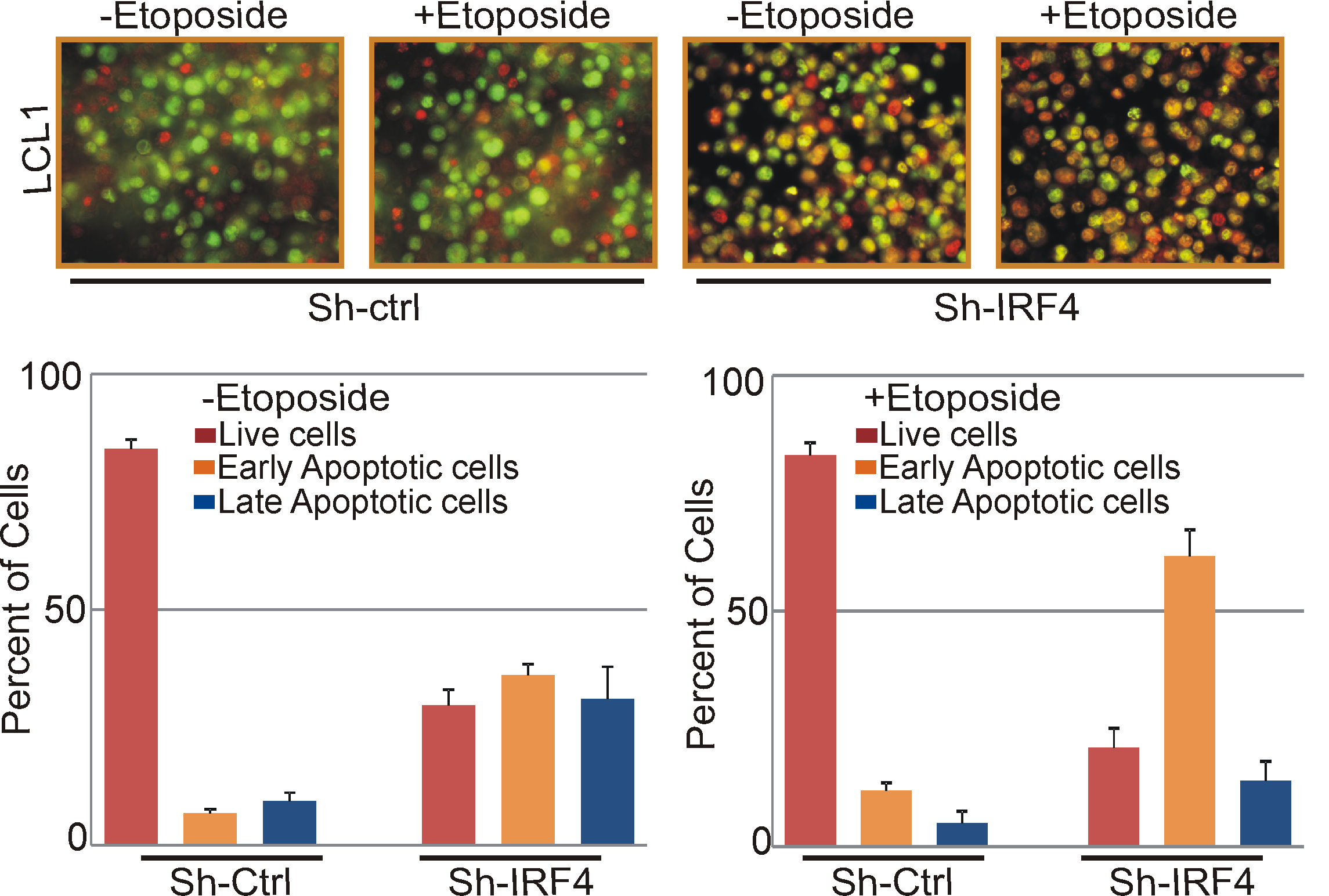

Supplement: Figure S6 — IRF4 knockdown enhances apoptosis in EBV transformed cells treated with etoposide. EBV transformed LCL1 cells were subjected to lentivirus mediated stable transduction by introducing short hairpin RNA (sh-RNA) to knockdown Irf4. Sh-Ctrl RNA also transduced for control set. Stable knockdown cells were treated with etoposide drug for different time points. Next, cells were harvested and pelleted by centrifugation at 1000 RPM (129 g) for 5 minutes. Cell pellets were washed with 1 ml of cold PBS and cell pellets were resuspended in 25 µl of cold PBS and 2 µl of EB/AO (ethidium bromide/acridine orange) dye mix. 10 µl of stained suspension were placed on clean slide and covered with coverslip. Cells were observed and counted by using fluorescence microscope [79]. Experiments were done in triplicates by counting a minimum of 100 total cells each. The data shown here indicates that etoposide treatment significantly enhanced the apoptosis in IRF4 knockdown stable EBV transformed LCL1 cells, compared with the control vector transfected cells. (TIF) [file ppat.1003314.s006.tif]

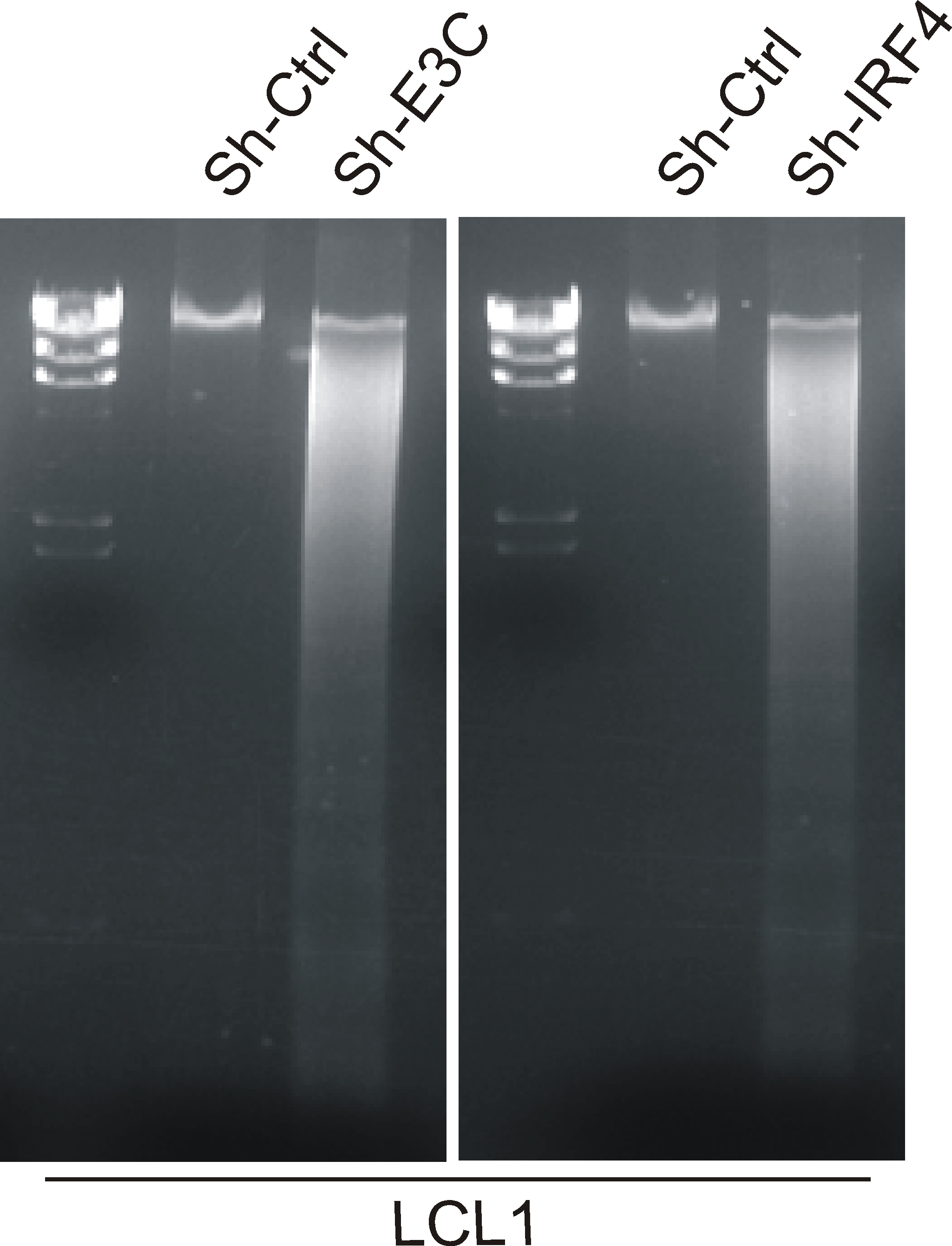

Supplement: Figure S7 — EBNA3C and IRF4 silencing promotes apoptotic induction in EBV transformed Lymphoblastoid cells. Apoptosis is potentially involved in regulation of cellular proliferation under a wide range of virus-induced pathogenic activities. Importantly, DNA fragmentation is one of the hallmarks of apoptosis [80]. In order to determine if apoptotic events contributed to the reduction in growth rate upon IRF4 knockdown cells, DNA fragmentation assay was performed. 4×10^6^ Sh-Ctrl, Sh-EBNA3C, Sh-IRF4 stable LCL1 cells were collected in 1.5 ml eppendorf tube after washing with 1X PBS Next, cell pellet re-suspend with 0.5 ml 1X PBS and 55 µl of Triton X-100 lysis buffer (40 ml of 0.5 M EDTA, 5 ml of 1 M trisCl buffer pH 8.0, 5 ml of 100% Triton X-100, 50 ml of H2O) was added for 20 min on ice. Tubes were centrifuged at 4°C at 12,000 g for 30 min. Samples were transferred to new tubes and supernatants was extracted by using 1∶1 mixture of phenol: chloroform. DNA precipitation was performed by adding in two equivalence of cold ethanol and one tenth equivalence of Sodium Acetate. DNA pellet was re-suspended with 30 µl of de-ionized water-RNase solution (0.4 ml of water with 5 µl of RNase solution) and 5 µl of loading buffer. Samples were incubated for 30 min for 37°C. DNA samples were run by 1.2% gel at 5 V for 5 min before increasing to 100 V. Interestingly, we observed a substantial amount of DNA fragmentation upon sh-RNA based knockdown of IRF4 and EBNA3C. (TIF) [file ppat.1003314.s007.tif]
