# Supplementary figures and images for: Expression of Concern: The EBV latent antigen 3C inhibits apoptosis through targeted regulation of interferon regulatory factors 4 and 8
Source: PLoS Pathog. 2025 Dec 16;21(12):e1013777. doi: 10.1371/journal.ppat.1013777 (PMC12707668; doi:10.1371/journal.ppat.1013777)

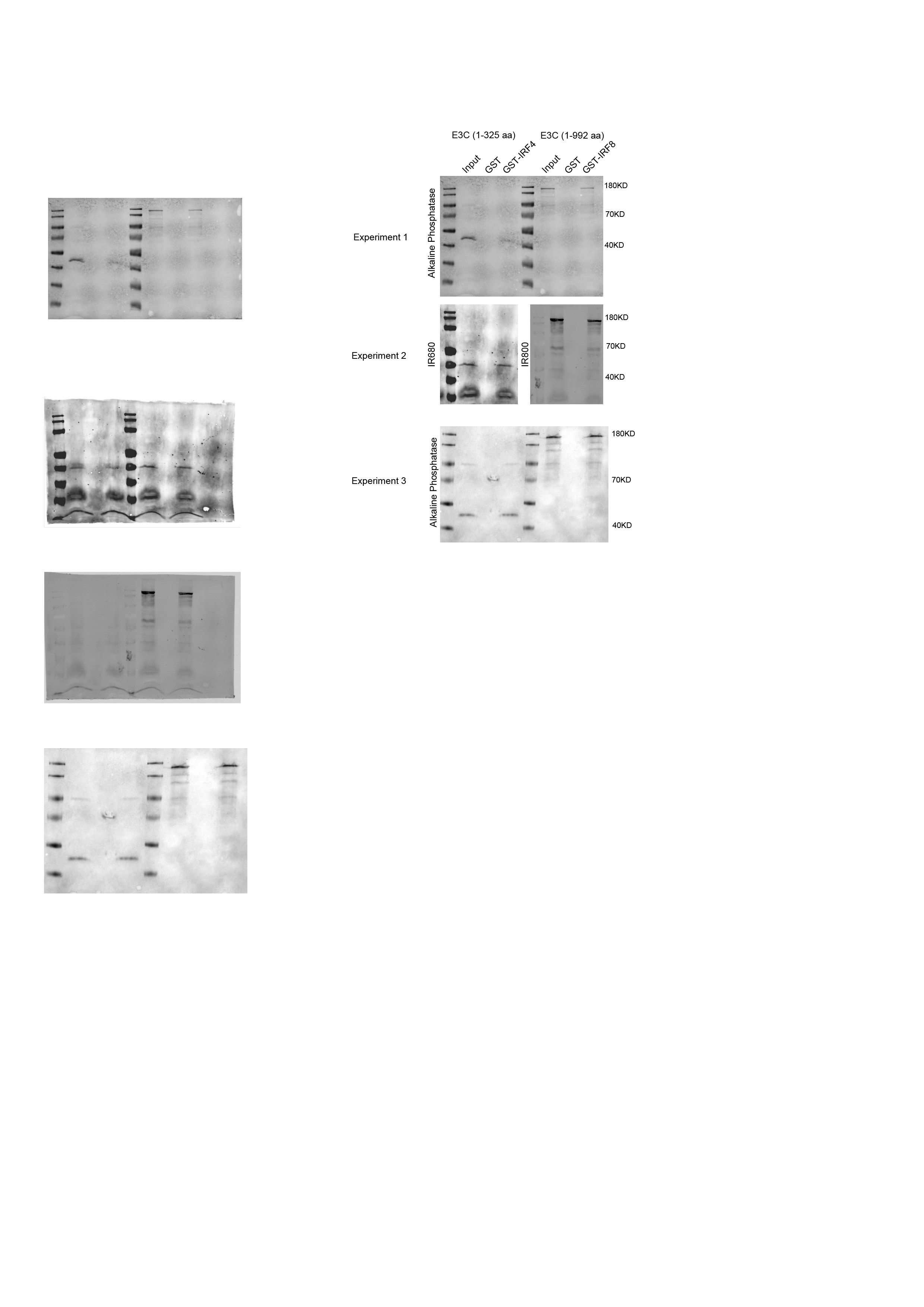

Supplement: S1 File — (JPG) [file ppat.1013777.s001.jpg]
