# Supplementary material for: Expression of Concern: The EBV latent antigen 3C inhibits apoptosis through targeted regulation of interferon regulatory factors 4 and 8
Source: PLoS Pathog. 2025 Dec 16;21(12):e1013777. doi: 10.1371/journal.ppat.1013777 (PMC12707668; doi:10.1371/journal.ppat.1013777)
Supplement: S2 File — (DOCX) [file ppat.1013777.s002.docx]

**IRF4 and IRF8 interaction with EBNA3C**

**Experiment:** IRF4 and IRF8 cloned in pGEX2 vector were transformed BL21 cells and were grown overnight and induced with 1 mM of IPTG for ON at 25˚C. The cells were lysed, and the total proteins were isolated. The GST tagged proteins were isolated using Sepharose beads.

0.5 µg of different EBNA3C plasmids (1-992 amino acid, 1-365 amino acid) were used for in vitro translation using biotinylated lysine.

Due to regulations, the authors did not use radioactive S35 for this experiment. They replaced S35 with biotinylated amino acid.

The biotinylated truncated EBNA3C were mixed with IRF4 or IRF8 GST fusion proteins (isolated from bacteria) and incubated for ON at 4˚C. The Sepharose beads were precipitated and washed with STE buffer for 3 times and were electrophoresed in 10% polyacrylamide gel and transferred to a membrane. The membrane was blotted with either Alkaline phosphatase (Experiment 1 and 3) or IR tagged (IR680 and IR 800) (Experiment 2) Streptavidin.
